# Versatile Photofunctionalization of Carbon Nanotubes via [4 + 2] Cycloaddition: A Facile Route to Hybrid Nanomaterials

**DOI:** 10.1002/smsc.202500191

**Published:** 2025-08-31

**Authors:** Giacomo De Crescenzo, Paula Sánchez‐Morena, Alberto Fraile, Matías Blanco, José Alemán

**Affiliations:** ^1^ Organic Chemistry Department Universidad Autónoma de Madrid 28049 Madrid Spain; ^2^ Institute for Advanced Research in Chemical Sciences (IAdChem) Universidad Autónoma de Madrid 28049 Madrid Spain; ^3^ Center for Innovation in Advanced Chemistry (ORFEO‐CINQA) Universidad Autónoma de Madrid 28049 Madrid Spain

**Keywords:** carbon nanotubes, covalent functionalization, hybrid materials, photocatalysis

## Abstract

The [4 + 2] photofunctionalization of carbon nanotubes of different characteristics, such as single‐walled carbon nanotubes (0.9 and 1.4 nm diameter) or multiwalled carbon nanotubes (10 nm diameter), with aryl cyclobutyl amines **3** of diverse nature in the presence of common organic photocatalysts is presented. Through a facile synthetic protocol, a series of modified amines **3** is prepared, which undergo a photocatalyzed oxidative ring opening to subsequently react with the nanotube wall. Under the best reaction conditions (Rhodamine B, white light‐emitting diode, 40 h), an 8–20% functionalization degree in the form of *N*‐cyclohexylamine moieties is achieved. The reaction is general with respect to the type of nanotube employed, the electronic characteristics of the amine (decorated with electron‐donating and electron‐withdrawing groups), and it is homogeneous throughout the whole nanotube particle without affecting the morphology of the samples, as demonstrated by the characterization techniques performed. In addition, the method allows for postfunctionalization treatments by the on‐surface hydrolysis of an ester‐modified aryl cyclobutyl amine and subsequent amidation with amino‐decorated organic polymer nanoparticles. Thus, a hybrid material with different properties compared to the simple composite is obtained using this photochemical carbon nanotubes functionalization.

## Graphical abstract

A [4 + 2] photocycloaddition of aryl cyclobutyl amines on carbon nanotubes is presented. A photocatalyst oxidizes the cyclic moiety to generate an active species trapped by the nanotube π‐cloud. The method functionalizes nanotubes of different characteristics and is tolerant to different functional groups decorating the amine. Postfunctionalization treatments can be performed to synthesis of hybrid materials by bonding nanotubes and organic polymers.

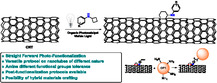

## 1 Introduction

Carbon nanotubes (CNTs) are carbon allotropes in which all atoms possess sp^2^ hybridization and form an aromatic honeycomb network rolled into cylinders of nanoscale diameter and variable length.^[^ ^1^ ^]^ CNTs can be classified as single‐walled carbon nanotubes (SWNTs)^[^ ^2^ ^]^ when they consist of a single‐carbon sheet, and multiwalled carbon nanotubes (MWCNTs) when multiple concentric carbon layers are rolled together and interact via van der Waals forces.^[^ ^3^ ^]^ Since their discovery by Iijima in 1991,^[^ ^4^ ^]^ a huge effort has been made to characterize the nature of these materials and to find applications aimed at improving and/or substituting classic systems.^[^ ^5^, ^6^ ^]^ In general, CNTs exhibit excellent electrical, electronic, and thermal conductivities, as well as impressive mechanical properties due to their intrinsic structure and the bonds established between carbon atoms.^[^ ^7^, ^8^, ^9^, ^10^ ^]^ Thus, CNTs find application in the design of (opto)electronic devices or in integrating composites for structural or energy‐related systems.^[^ ^11^, ^12^, ^13^, ^14^ ^]^ Their carbon nature makes them suitable candidates for bioapplications,^[^ ^15^ ^]^ and their large surface area,^[^ ^16^ ^]^ combined with all these properties, has been exploited for catalysis purposes.^[^ ^17^, ^18^, ^19^, ^20^, ^21^, ^22^, ^23^ ^]^

**Scheme 1:**
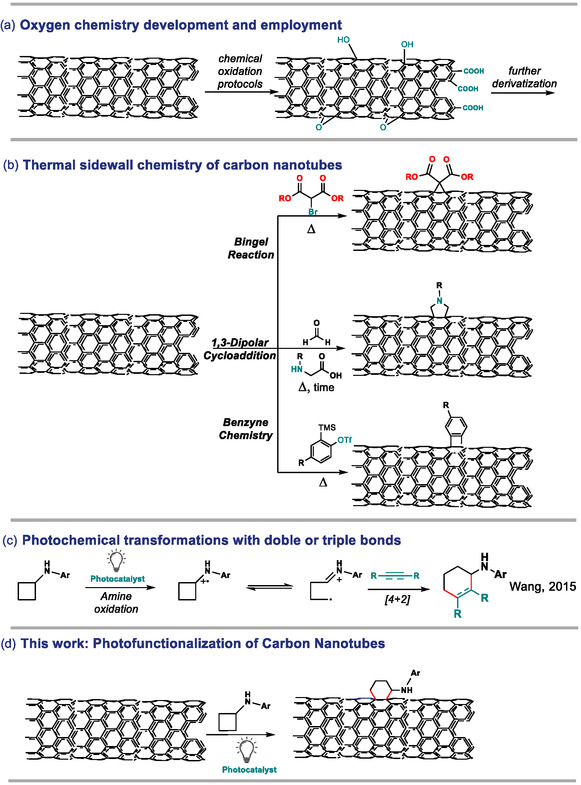
a) Development of oxygen chemistry on the surface of the nanotubes. b) Diverse strategies reported for the covalent functionalization of CNTs by cycloaddition reactions. c) Photochemical activation of double and triple bonds with cyclic amines. d) This work.

The chemistry of CNTs has also been studied.^[^ ^24^ ^]^ On the one hand, pristine CNTs can interact by supramolecular recognition with aromatic targets through reversible π–π or ionic interactions.^[^ ^25^, ^26^, ^27^ ^]^ Mechanical bonds can also be set in order to supramolecularly modify CNT properties.^[^ ^28^ ^]^ In addition, the encapsulation of organic and inorganic units in their inner cavity also represents another noncovalent functionalization of unaltered CNTs.^[^ ^29^, ^30^, ^31^ ^]^ On the other hand, covalent modifications of CNTs can also be performed. First, their carbonaceous structure is suitable for controlled oxidation reactions. Heating under air, introducing the material into reactive plasmas, or exposing the CNTs to different acids provoke the development of the oxygen surface chemistry^[^ ^32^, ^33^, ^34^ ^]^: Carboxylic acids on the edges and hydroxyl‐epoxy groups on the basal plane of the nanotube are typically formed upon oxidation and can be exploited for further functionalizations (**Scheme** **1**a), thus giving access to a wide gamut of modified materials with different properties.^[^ ^21^ ^]^ Furthermore, the carbon skeleton may also be employed as a reactive center for covalent functionalization, as the electron‐rich conjugated aromatic system can interact with different molecules or moieties. A particularly common reaction pathway is the cycloaddition one. For instance, cyclopropanation reactions (known as the Bingel reaction)^[^ ^35^ ^]^ between a bromomalonate and CNTs in the presence of a base and refluxing conditions have been reported (top Scheme 1b). Furthermore, the Diels–Alder reaction or the 1,3‐dipolar cycloaddition of azomethine ylides with CNTs,^[^ ^36^, ^37^, ^38^ ^]^ which are generated by the reaction of aminoacids and aldehydes for several days at high temperatures, are well‐known functionalization strategies (middle Scheme 1b). Moreover, benzyne generation through basic elimination conditions, both thermal or under microwave activation, has been reported as a useful method for CNT functionalization within cycloaddition methodologies (bottom Scheme 1b).^[^ ^39^, ^40^ ^]^ Nevertheless, the development of visible light‐driven modification of CNTs is still very unexplored, even though some reports with UV illumination can be found.^[^ ^41^, ^42^, ^43^ ^]^

In this sense, photochemical transformations represent a mild and sustainable strategy to achieve a chemical reaction by converting the energy of light into a chemical vector.^[^ ^44^ ^]^ Rather than using very energetic radiation such as UV, one of the most efficient strategies entails the employment of a photocatalyst, as they can often interact with visible light.^[^ ^45^ ^]^ Photocatalysts allow triggering reactivities that would otherwise be very slow or impossible to achieve in their absence. For instance, the photocatalyzed oxidation of aliphatic amines to the corresponding amine radical cation is a known reaction, which can be employed both as a sacrificial closing step of a catalytic cycle and for reactive purposes, such as a [4 + 2] cycloaddition with unsaturated molecules employing aryl cyclobutyl amines (Scheme 1c).^[^ ^46^ ^]^ Since this reactivity has been designed for the functionalization of double and triple bonds in organic synthesis, we envision that similar mechanisms might take place when this chemistry is performed with CNTs (Scheme 1d).

In this work, we present a light‐driven functionalization strategy of CNTs through the photo‐oxidation of aryl cyclobutyl amines to promote a [4 + 2] cycloaddition with the double bonds located on the surface of CNTs. We explore the generality of the method by employing different aryl cyclobutyl amines via distonic radical cations, bearing donor, withdrawing, and reactive functional groups, to react with CNTs of different characteristics (SWNTs of different diameters and MWNTs). As a result, highly modified hybrids, compared to pristine surfaces, are obtained, bearing different functionalities, including reactive ones, that are anchored by a cyclohexylamino motif. Indeed, we fully characterize all the functionalized materials by spectroscopic and microscopic techniques to unveil the nature of the newly created covalent bonds, opening chemical pathways for the construction of hybrid materials by covalent bonding with polymer particles.

## 2 Results and Discussion

**Scheme 2:**
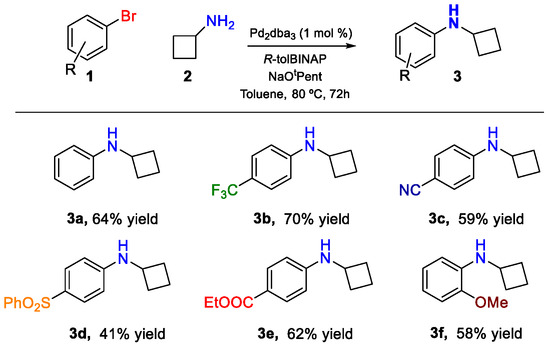
Synthesis of aryl cyclobutyl amines **3**.

We started our study with the synthesis of the precursors to be submitted to the functionalization (see **Scheme** **2** and Table S1 at Supporting Information for full details on the synthesis and characterization of the starting materials). Thus, aryl cyclobutyl amines **3** were produced with good yields by reaction of cyclobutyl amine with the bromoaryl derivatives through a palladium‐catalyzed Buchwald–Hartwig cross‐coupling protocol, as depicted in Scheme 2.^[^ ^46^ ^]^ In particular, we were able to synthesize aryl cyclobutyl amines decorated with different substituents, such as phenyl cyclobutyl amine (**3a**), in 64% isolated yield after 3 days of reaction. In addition, decorated aryl cyclobutyl amines were also synthesized, such as the *p*‐trifluoromethylphenyl cyclobutylamine (**3b**), the *p*‐cyanophenyl derivative **3c**, or the *p*‐phenylsulfonyl‐benzene example **3d**, which were obtained in 70%, 59%, and 41% yields, respectively. Furthermore, ethyl benzoate cyclobutyl aniline (**3e**), which can be used for further derivatizations, could also be yielded in 62%. Even more, an electron‐rich amine such as *o*‐methoxyphenyl cyclobutylamine (**3f**) was also yielded under the same procedure in a moderate 58% yield (see Scheme 2).

After the synthesis of aryl cyclobutyl amines, we started to screen the reactivity of these substrates towards a potential [4 + 2] reaction with the aromatic network of CNTs, using *N*,*N*‐cyclobutylphenyl amine **3a** as a model reaction. Initially, we mimicked the conditions described for the homogeneous reaction with aryl cyclobutyl amines,^[^ ^46^ ^]^ using the iridium complex [Ir(dtbbpy)(ppy)_2_]PF_6_ (dtbbpy = 4,4′‐Di‐*tert*‐butyl‐2,2′‐dipyridyl; ppy = 2‐phenylpyridine) as the photocatalyst in *o*‐dichlorobenzene to avoid introducing any N source from the solvent for analytical purposes. After 40 h of visible light illumination (white light‐emitting diode (LED)), under an inert atmosphere and using three different types of carbon nanotubes (SWNT of 0.9 nm, SWNT of 1.4 nm, and MWNT of 10 nm), we obtained black solids, which were cleaned by filtration–sonication methods (see Experimental Section). To our delight, we detected an increase in the N content determined by elemental analysis in samples **SWNT‐09‐3a** (0.9%), **SWNT‐14‐3a** (0.7%), and **MWNT‐3a** (0.9%) compared to the pristine nanotubes (0.3%, 0.4%, and 0.1%, respectively). This increase may be caused by the introduction of the amine after the photoreaction (see Table S2, Supporting Information for full elemental analysis). Motivated by this promising result, we started the optimization of the reaction employing cyclobutyl phenyl amine (**3a**) and **SWNT‐09** as model substrates. The results are depicted in **Table** **1** as the differential content of nitrogen between the experiment and the initial N content of the parent nanotube. First, we performed control experiments to confirm the nature of the functionalization. On the one hand, the reaction required light to proceed, because the control in the absence of light afforded 0.4% N, which is very similar to the content of the parent sample **SWNT‐09** (0.1% ΔN amount, Table 1, entry 2). However, the reaction seemed to proceed to a small extent without the presence of the iridium photocatalyst, because we obtained a similar N content in the elemental analysis of that sample compared to the initial experiment (0.3% ΔN amount, Table 1, entry 3). This result made us consider whether there is a mismatch in the redox potentials of the catalyst and substrate **3a**. Indeed, the oxidation potential of **3a** was reported to be 0.8 V versus saturated calomel electrode (SCE),^[^ ^46^ ^]^ while the reduction potential of the photoexcited Ir photocatalyst was determined as 0.66 V versus SCE (a reaction using phenyl acetylene as substrate under our experimental conditions resulted in decomposition of the substrates, Table 1, entry 4, also determined by ^1^H‐NMR).

**Table 1:** Model [4 + 2] photocycloaddition of *N*,*N*‐cyclobutylphenyl amine **3a** over **SWNT‐09** sample.

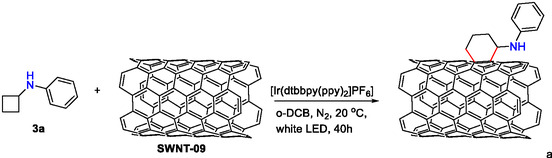		
Entrya)	Variation from the standard conditions	%ΔNb)
1	No variation	0.6
2	No light	0.1
3	No photocatalyst	0.3
4	Unsaturated substrate = Ph‐≡‐H instead of **SWNT‐09**	0c)
5	Photocatalyst = Eosin Y	1.6
6	Photocatalyst = Rose Bengal	0.9
7	Photocatalyst = Rhodamine 6 G	1.8
8	Photocatalyst = Rhodamine B	2.0
9	Nanotube = SWNT‐14 with Rhodamine B	1.0
10	Nanotube = MWNT with Rhodamine B	1.2

a) Reaction conditions: 0.1 mmol of substrate, 0.01 mmol of photocatalyst, 3 mg of nanotubes in 1.5 mL of *o*‐DCB, under inert atmosphere with white LED illumination for 40 h.

b) Values reported in %wt.

c) yield (%).

To better align the potentials and remove the metals from our reaction, we tested other organic photocatalysts, such as Eosin Y (*E* = 0.83 V vs. SCE), Rose Bengal (*E* = 0.81 V vs. SCE), Rhodamine 6G (*E* = 0.95 V vs. SCE), and Rhodamine B (*E* = 0.84 V vs. SCE).^[^ ^45^ ^]^ The better alignment of Eosin Y (1.6% ΔN, Table 1, entry 5), Rhodamine 6G (1.8% ΔN, Table 1, entry 7), and Rhodamine B (2.0% ΔN, Table 1, entry 8) resulted in higher incorporation of N onto the surface of the nanotubes after illumination compared to the previous Ir catalyst. On the other hand, Rose Bengal struggled somewhat during the oxidation of the amine to initiate the photocycloaddition (0.9% ΔN, Table 1, entry 6). Therefore, using the best photocatalyst, Rhodamine B, we performed the reaction with the other nanotubes, obtaining very good results. Hence, sample **SWNT‐14‐3a** was functionalized with a 1.0% incorporation of N, while sample **MWNT‐3a** presented 1.2% ΔN according to the elemental analysis determinations, highlighting the generality of the method. It must be noted that the best nanotubes to perform the [4 + 2] photofunctionalization appeared to be sample **SWNT‐09**. It is important to mention that neither the solvents employed nor the organic photocatalyst contained N atoms, indicating that all the N detected under these optimum conditions was due to the incorporation of the aryl cyclobutyl amine. The degree of functionalization of these samples was also analyzed by thermogravimetric analysis (TGA), achieving weight losses of 14%, 12%, and 8% at temperatures around 350–400 °C (typical temperature for functionalization analysis according to the literature)^[^ ^47^ ^]^ for samples **SWNT‐09‐3a**, **SWNT‐14‐3a**, and **MWNT‐3a**, respectively (Figure S3, Supporting Information). These results matched the determinations performed by elemental analysis (see S.I. for further details on the functionalization degree).

**Figure 1:**
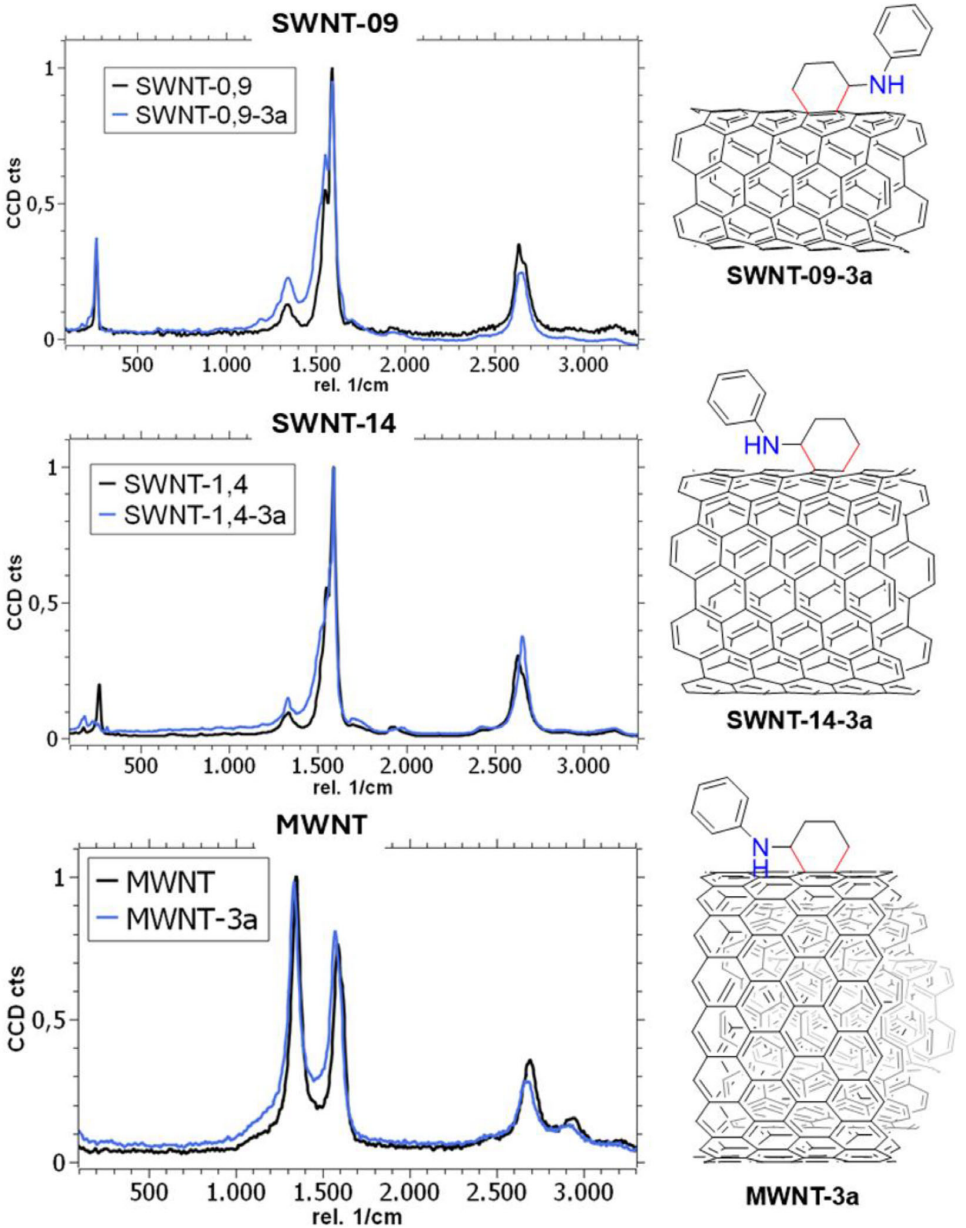
Raman spectra of nanotube samples **SWNT‐09**, **SWNT‐14**, and **MWNT** before (black line) and after the [4 + 2] photofunctionalization with cyclobutylaniline (**3a**) (blue line), including plausible structures.

The functionalization was also monitored by Raman spectroscopy. This analytical technique represents a powerful method to characterize carbon nanomaterials, particularly CNTs, and to assess whether a functionalization treatment has affected the structure of the nanomaterial. Even considering that we have different types of CNTs, a general Raman pattern consists of three distinctive bands: the sp^2^ in‐plane vibrations, also noted as the “graphitic” or G band (≈1590 cm^−1^); the dislocation vibrations related to structural defects, referred as the “disorder” or D band (≈1340 cm^−1^); and the overtone of the G band, known as the 2D band (≈2650 cm^−1^). In addition, the breathing vibration of the tube (radial breathing mode, RBM), related to the diameter of the sample, can be observed for SWNTs in the region of ≈180–200 cm^−1^.^[^ ^48^ ^]^ This general pattern was observed for parent **SWNT‐09**, **SWNT‐14**, and **MWNT** samples (**Figure** **1**). The structural integrity of these materials is usually assessed using the ratio of the relative intensities of the graphitic and defect bands (*I*_D_/*I*_G_), presenting sample values of 0.07, 0.07, and 1.19 for **SWNT‐09**, **SWNT‐14**, and **MWNT**, respectively. After illumination under the best conditions, using cyclobutyl phenyl amine (**3a**) as the substrate for the [4 + 2] photocycloaddition, we observed an increase in the intensity of the D band in the Raman spectra for all samples. The increase in defect bands is regarded in the literature as a positive indicator of functionalization. Indeed, sample **SWNT‐09‐3a** presented an *I*_D_/*I*_G_ value of 0.20 while maintaining a constant RBM band position, indicating that the functionalization occurred only at the outer wall of the nanotube. It is noteworthy that the G band shifted 3 cm^−1^ to the blue, suggesting that after the treatment, the material was doped due to the presence of electron‐rich heteroatoms attached to the material (Figure S4, Supporting Information). Similar results were obtained for samples **SWNT‐14‐3a** and **MWNT‐3a**, with *I*_D_/*I*_G_ ratios increasing to 0.12 and 1.26, respectively, while the rest of their corresponding Raman features remained unaltered. They also presented similar G band shifts. Therefore, the Raman data suggest that the increase in N detected by elemental analysis may result from functionalization of the CNT walls, and different CNTs may have followed a similar trend regarding the extent of functionalization. Furthermore, comparable *I*_D_/*I*_G_ increments have been previously reported for successful functionalization treatments.^[^ ^49^ ^]^

In addition, the Fourier transformed infrared (FTIR) spectra of these samples exhibited two scissoring vibrations at ≈2860 cm^−1^ and ≈2920 cm^−1^ (Figure S5, Supporting Information), although in some cases they were difficult to observe, possibly due to intrinsic sensitivity limitations of the technique. These bands are characteristic of aliphatic C—H bonds, reflecting the formation of the six‐membered ring between the aliphatic amine and the nanotube. Thus, new bonds that modify the surface of the nanotubes are created by this photocatalytic method.

**Figure 2:**
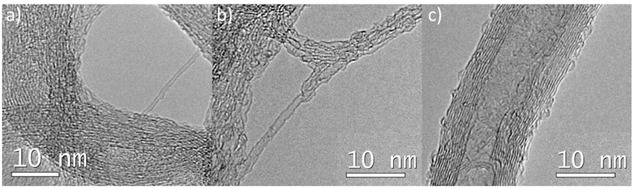
TEM images of samples: a) **SWNT‐09‐3a**, b) **SWNT‐14‐3a**, and c) **MWNT‐3a**.

The structural integrity was also checked by high resolution transmission electron microscopy (**Figure** **2**). The analysis of the samples determined that the structural morphology of the nanotubes was preserved after illumination, being very similar to the pristine nanotubes (Figure S6,S7, Supporting Information). Smooth tubes packed in arrays were observed for each kind of material, while some protrusion, rupture, or deformation was detected for samples **SWNT‐09‐3a**, **SWNT‐14‐3a**, and **MWNT‐3a** after exhaustive analysis and comparison with the pristine materials (agreeing with external wall functionalization in the [4 + 2] photocycloaddition).^[^ ^50^ ^]^ Furthermore, the presence of N was detected in the in situ energy‐dispersive X‐ray (EDX) spectra of these samples (Figure S8, Supporting Information), which may confirm the introduction of the amine moiety as a covalent modifier of the nanotubes as a result of the performed photoreaction.

**Figure 3:**
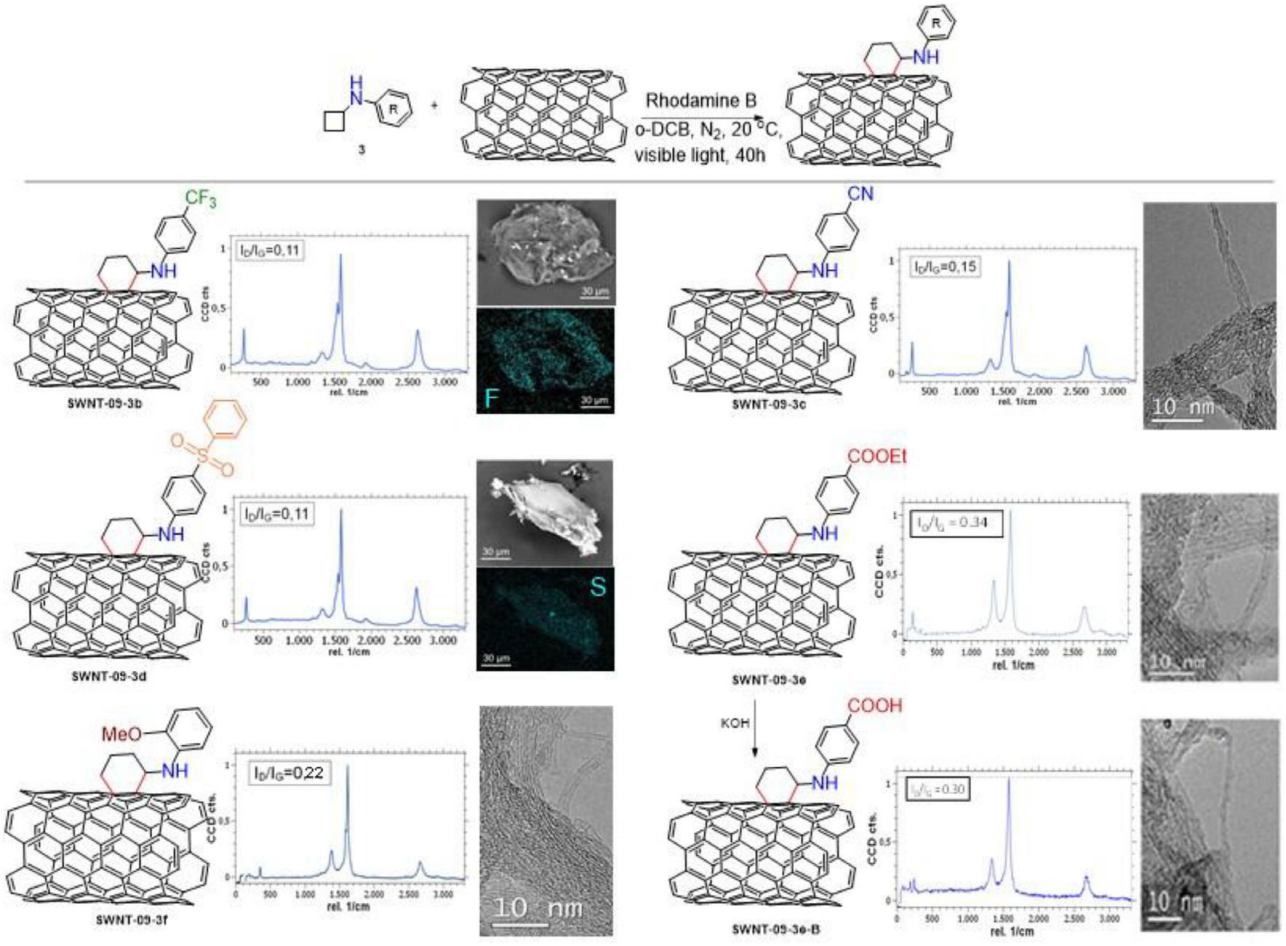
Scope of the [4 + 2] photocycloaddition on the organic cyclobutyl anilines.

After confirming that the functionalization can be carried out with different nanotubes using the model cyclobutyl phenyl amine (**3a**), we modified the characteristics of the organic substrates to be functionalized on the surface of the nanotube to expand the scope of the method (**Figure** **3**). Thus, we added different groups which modified the electronics of the substrate, as well as heteroatoms that could be easily identified with the analysis techniques. These reactions were performed only with the **SWNT‐09** nanotubes sample, and the results are summarized in Figure 3 and fully shown in S.I. Indeed, positive functionalization was spotted in the elemental analysis since all the samples possessed higher contents of N compared to the pristine nanotube (see Table S3, Supporting Information). Specifically, sample **SWNT‐09‐3b** presented a 1.4 wt% of N, matching the weight loss at 350–400 °C in TGA of 14% (see Figure S9, Supporting Information), while the *I*_D_/*I*_G_ ratio was calculated as 0.11. It can be seen that this sample presented higher values compared to pristine nanotubes **SWNT‐09**. We also observed homogeneous functionalization throughout the arrays of tubes (where morphological aspects seemed very similar to the pristine sample **SWNT‐09**, Figure S7, Supporting Information), since the scanning electron microscopy (SEM)–EDX mapping detected the presence of F corresponding to the CF_3_ all along the analyzed particle. A closer analysis by transmission electron microscopy (TEM)–EDX also detected the fluorine signal, thus confirming that the F‐functionalized nanotubes were only responsible for emitting F signals in the EDX spectrum (see Figure S11, Supporting Information). Other electron‐withdrawing groups, such as a nitrile or phenyl sulfone, were also tolerated. Indeed, sample **SWNT‐09‐3c**, which contained a nitrile group, showed 2.2% of N in the elemental analysis (higher than the previous samples due to the presence of the nitrile group). The nitrile could be observed in the FTIR (Figure S5, Supporting Information),^[^ ^51^ ^]^ and the sample presented an *I*_D_/*I*_G_ ratio of 0.15. Conversely, sample **SWNT‐09‐3d**, which contains the sulfone moiety, showed 1.3 wt% of N and 1.5 wt% of S (crosschecked in the TGA with 13% and 16% weight loss, respectively). This sample was the only one to present a higher than 1 S/N ratio (see Table S3, Supporting Information). Sulfur was also spotted homogeneously distributed over the array particle and locally in the TEM–EDX analysis (Figure S11, Supporting Information), and the *I*_D_/*I*_G_ ratio was calculated as 0.11.

Furthermore, an electron‐rich substrate such as the *o*‐methoxyphenyl cyclobutylamine (**3f**), which is more prone to get oxidized than the electron‐poor amines **3b‐e**, also worked well in the [4 + 2] photocycloaddition. Hence, a 1.3 wt% of N (10% weight loss by TGA) was detected in the elemental analysis, and the *I*_D_/*I*_G_ ratio was observed to be 0.22, in agreement with the electronic character of this particular substrate. Finally, a different electron‐withdrawing group such as the ethyl ester of substrate **3e** was also checked. This sample, **SWNT‐09‐3e**, was found to have been functionalized to a greater extent, with a very modified structure as a result of the treatment (*I*_D_/*I*_G_ = 0.34, in agreement with some apparent disruption at the nanotube walls detected by TEM), showing a 1.8 wt% of N value measured by elemental analysis. This ester was hydrolyzed with KOH, forming the carboxylic acid (see Scheme S2, Supporting Information). Indeed, the FTIR spectra showed a slight shift in the carbonyl band (from 1730 cm^−1^ to 1718 cm^−1^ after the hydrolysis), a decrease in the intensity of the C—H signals, and a modification of the fingerprint region of the hydrolyzed sample (Figure S5, Supporting Information).^[^ ^52^ ^]^ In addition, the nitrogen content and the chemical structure appeared approximately unmodified (*I*_D_/*I*_G_ = 0.30, 1.6% N, see Table S3, Supporting Information) according to TEM, Raman, and elemental analysis determinations. It is worthy to mention that the weight loss curve slope of this sample appeared less pronounced since the decorating moiety had lost the ethoxy group as a result of the hydrolysis reaction, which implies less weight loss at the TGA assay. Indeed, the basic hydrolysis seems to have only affected the ester group, respecting both the nanotube structure and the cyclohexyl moiety, which is essential for a postfunctionalization treatment. In summary, a wide variety of functions present in the modifying molecule, including reactive ones that allow postfunctionalization, can be smoothly introduced at the surface of the CNTs under mild photochemical conditions.

**Figure 4:**
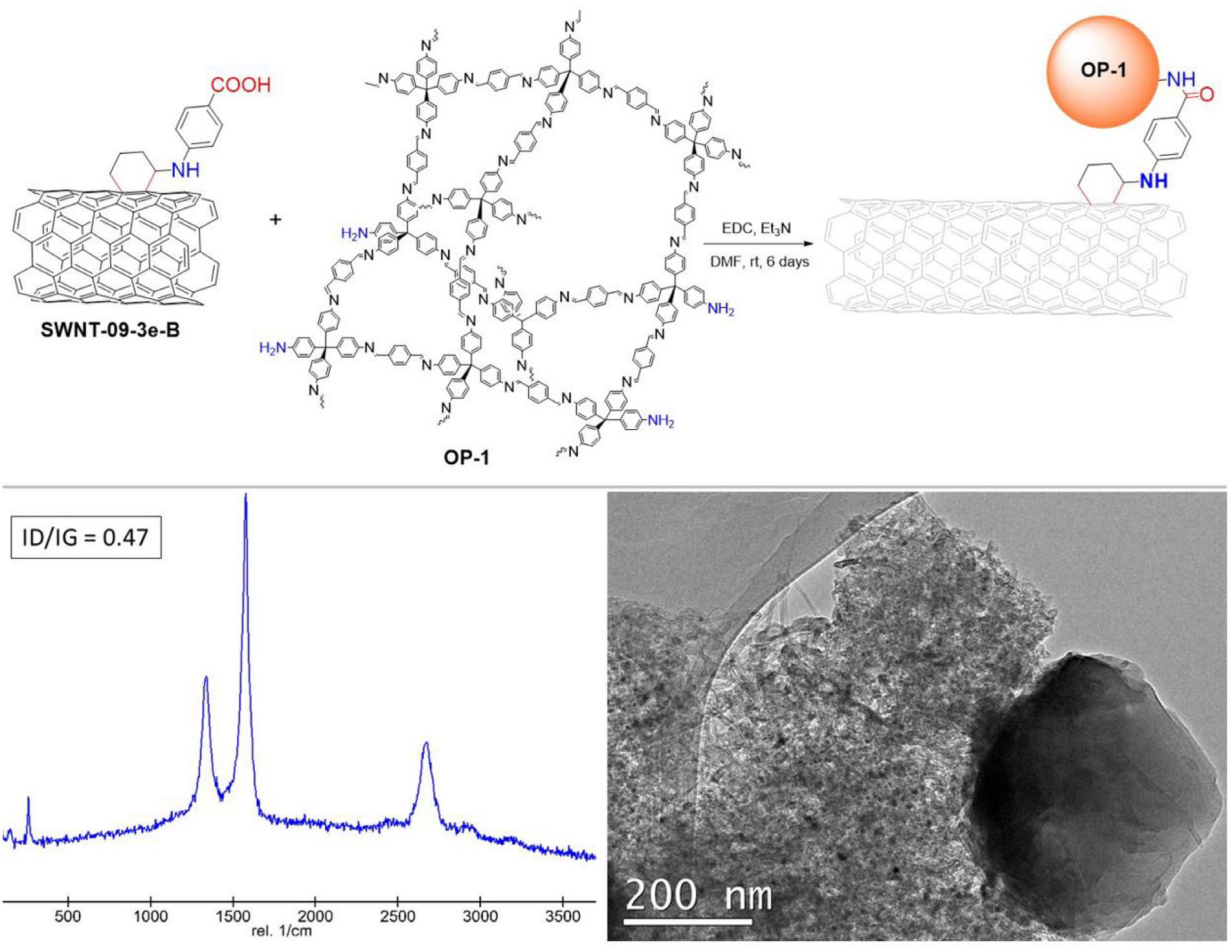
Synthesis of the hybrid material **SWNT‐OP‐1** formation with the Raman and the TEM characterization.

In order to expand the applicability of the photofunctionalization method, we constructed a hybrid material by covalent methodology, combining the hydrolyzed sample **SWNT‐09‐3e‐B** with an amino‐enriched imine‐based organic polymer (**OP‐1**). **OP‐1** was synthesized by the reaction of tetra(4‐anilyl)methane and substoichiometric amounts of terephtaldehyde (see SI for further details),^[^ ^53^ ^]^ slightly modifying a procedure previously reported (see SI for full characterization of the material).^[^ ^54^ ^]^ The defective amount of aldehyde during polymerization afforded ≈100 nm‐diameter sphere‐like material that contained defects as free amino groups detected by SEM and FTIR, respectively (especially with a band at ≈3200 cm^−1^ corresponding to the N—H bond vibration, see Figure S13, Supporting Information). Moreover, the sample presented the typical profile in the TGA for this kind of material, characterized by a high N and H content observed in the elemental analysis as a result of the properties of its constituents. The solid‐state ^13^C‐NMR analysis of the polymer (Figure S15, Supporting Information) revealed the presence of aromatic and aliphatic carbon atoms, including the iminic polymerization bond observed at 160 ppm.^[^ ^54^ ^]^ The pending reactive moiety could be employed in an amidation coupling through carbodiimide chemistry (**Figure** **4**).^[^ ^55^ ^]^ As a result, a hybrid material, **SWNT‐OP‐1**, could be yielded, where some of the nanotubes were covalently bound by amide bonds to the spheres of the organic polymer.

With a simple optical view at the confocal microscope, the morphological aspect of the hybrid material greatly differs from the starting organic polymer. Indeed, the bare **OP‐1** appeared as an almost fluorescent, globular‐grouped yellow material (Figure S14,S16, Supporting Information). The addition of nonfunctionalized nanotubes to the polymer creates a heterogeneous composite where distinguishing both components becomes an easy task. However, the hybrid material presented a different aspect, where the nanotubes shine very similarly to the functionalized ones, perhaps due to the covalent functionalization between nanotubes and polymer particles. In addition, the Raman spectrum of this hybrid material showed the typical features of both **SWNT** and **OP‐1**, but in this case, the D band of the polymer appeared as a tiny shoulder, redshifted from 1152 cm^−1^ in the pristine polymer to 1166 cm^−1^ in the hybrid material (see Figure S14, Supporting Information). This redshift might indicate a successful formation of the amide bond due to charge transfer phenomena between partners through the newly created bonds,^[^ ^56^ ^]^ which is accompanied by an increase in the *I*_D_/*I*_G_ ratio of the nanotube structure from 0.3 to 0.47. Indeed, the FTIR spectrum of the hybrid **SWNT‐OP‐1** presented a carbonyl band centered at 1700 cm^−1^,^[^ ^57^ ^]^ which is an indicative position of amide bonds (and different from the band of a typical carboxylic acid at a higher wavenumber), while the presence of the amine band could not be detected, and a high content of H and N (3.7% and 2.3%, respectively) was observed by elemental analysis as a result of the polymer bonding. In addition, the thermal stability of the hybrid material was deeply modified,^[^ ^58^ ^]^ initiating the weight loss at temperatures around 200 °C (Figure S12, Supporting Information), presenting a profile with an intermediate shape between the polymer and the nanotube as a result of the applied treatment. On the contrary, the thermal profile of a physical mixture of both constituents was completely different from the hybrid composite. Indeed, this TGA resembled the plots of pristine materials (see Figure S12, Supporting Information).^[^ ^59^, ^60^, ^61^ ^]^ Since all the features suggest the formation of the covalent bond between the nanotube and the polymer, the hybrid material **SWNT‐OP‐1** was morphologically analyzed by means of TEM imaging and compared to its components **SWNT‐09‐3e‐B** and **OP‐1**. The composite without the chemical bond between the nanotubes and the polymer particles was analyzed as a control. Hence, an intimate contact between the polymeric material and the nanotube bundles was systematically observed after exhaustive TEM observations for sample **SWNT‐OP‐1** (Figure 4 and S18, Supporting Information), and in some cases, **OP‐1** seemed completely embedded in the nanotube matrix, with particles coalesced to a small extent. This fact might explain the observed optical aspect discussed above. However, it was not possible to observe either isolated polymer nanoparticles or individual nanotube arrays. Furthermore, the nanotubes and the polymer particles presented a veiled aspect, perhaps due to partial disaggregation of the polymer as a result of the treatment. Conversely, the physical mixture of nanotubes and polymer without any chemical bond presented a very different aspect. Indeed, in some regions of the TEM grid, individual aggregates of nanotubes were spotted, while other regions showed lone and unaltered polymer particles. In the best cases, both components were observed close but not in contact (see Figure S19, Supporting Information), thus highlighting the need for a chemical bond, in the form of an amide, to create a hybrid material with different properties compared to the individual counterparts and therefore validating the [4 + 2] light‐mediated functionalization method.

## 3 Conclusion

This work describes a method for the covalent functionalization of carbon nanotubes with aryl cyclobutyl amines using a photocatalytic approach. The protocol takes advantage of the photo‐oxidative ring opening of the cyclobutyl moiety in the presence of an organic photocatalyst and white LED illumination to generate an active species able to react with the nanotube wall. As a result, cyclohexyl‐amino motifs decorate the CNT without compromising the structure and the general properties of the nanomaterial, using different types of nanotubes. Indeed, SWNT of 0.9 and 1.4 nm and MWNT of 10 nm could be functionalized in a range of 8%–20%, with the most reactive sample being **SWNT‐09** under our experimental conditions. In addition, the functionalization treatment is general with different aryl cyclobutyl amines bearing electron‐donating and electron‐withdrawing groups. In all cases, we observed smooth and homogeneous functionalization throughout the whole nanotube particle, thus validating the method. As a result of the investigation, we discovered that a wide variety of functionalities present in the modifying molecule can be introduced at the surface of the CNTs by a mild photochemical method. Indeed, aryl cyclobutyl amines with different characteristics, including reactive ones, can be effectively grafted to the nanotube surface, representing an advantage due to surface modification compared to the pristine materials. The method can be considered a versatile modification tool for CNTs, applying for the first time photochemical principles to functionalization. Finally, the applicability of the method was studied on postfunctionalization treatments. We performed the on‐surface hydrolysis of an ester‐modified aryl cyclobutyl amine, forming the carboxylic acid, which was amidated with amino‐decorated organic polymer nanoparticles. While the basic hydrolysis only affected the ester group, the hybrid material yielded differed from the isolated components both morphologically and spectroscopically. We believe that this method paves the way for the employment of light‐mediated nanocarbon functionalization protocols and for the synthesis of hybrid materials using light as a driving force.

## 4 Experimental Section

4.1

4.1.1

##### General Information. Materials and Methods

All chemicals, solvents, and reagents were purchased from commercial sources (reagent grade quality or better) and used without further purification if not otherwise stated. SWNT of 0.9 nm and 1.4 nm in diameter were purchased from Aldrich and purified by HCl treatment as previously reported (see Supporting Information, S.I.).^[^ ^29^ ^]^ MWNT were purchased from Nanocyl and employed as received. The purification of organic products, when necessary, was accomplished by flash chromatography using silica gel (Merck Geduran Si 60) in an adequate mixture of cyclohexane (CyH) and ethyl acetate (EtOAc) eluents. All the organic products were characterized by a comparison of their spectral data with those reported in the literature, from commercial sources or fully described. The synthesis of the aryl cyclobutyl amines **3** is fully described in the S.I. The synthesis of the organic polymer **OP‐1** and its characterization is depicted in the S.I. The postfunctionalization hydrolysis and the carbodiimide coupling protocols are also fully described in the S.I.

Nuclear magnetic resonance (NMR) spectra were obtained using a BRUKER AVANCE spectrometer running at 300 MHz for ^1^H and 75 MHz for ^13^C or in a BRUKER AV‐500 running at 500 MHz for ^1^H and 125 MHz for ^13^C. Spectra were internally referenced to the residual CDCl_3_ signal: *δ* 7.26 ppm for ^1^H‐NMR. Data for ^1^H‐NMR were reported as follows: chemical shift (*δ* ppm), multiplicity, coupling constant *J* (Hz), and integration. Solid‐state nuclear magnetic resonance (SSNMR) were recorded at a frequency of 100.61 MHz (9.4 T) and 376.49 MHz (9.4 T), respectively, with a Bruker AV400 SSNMR spectrometer using a 2.5 mm double‐resonance magic angle spinning (MAS) probe at a spinning speed of 10 kHz. To avoid baseline distortions, a rotor‐synchronous echo sequence (τ_R_‐π‐τ_R_) was applied prior to signal acquisition, where *τ*_R_ denotes one rotor cycle. The π‐ and π/2‐pulse widths for ^13^C were 6.0 μs and 3 μs, respectively. Recycle delays of 20 s were used. Resonance positions were referenced with respect to tetramethylsilane (TMS) using the CH_2_ resonance of adamantane at 38.56 ppm as a secondary reference. Electrospray ionization mass spectra (ESI‐MS) were obtained on an Agilent Technologies 6120 Quadrupole LC/MS coupled with a Supercritical Fluid Chromatograph (SFC) Agilent Technologies 1260 Infinity Series instrument. TEM images were obtained using a JEOL–JEM 2100 F instrument operating in high‐vacuum conditions (below 10^−5^ mbar) at an accelerating voltage of 200 KV, equipped with a liquid‐N_2_ cooled CCD high resolution camera and an Oxford EDX spectrometer in situ microprobe. Samples were drop casted from nanotube methanol suspensions on holey‐carbon copper grids. For the elemental analysis measurements, a LECO CHNS‐932 Analyser (Model NO: 601‐800‐500) was used. SEM images were carried out on a Hitachi S‐3000 N electron microscope with a coupled ESED detector and an analyzer from energy‐dispersive X‐ray from Oxford Instruments, INCAx‐sight model. TGA measurements were carried out on a thermobalance TGA Q500 from TA Instruments with a ramp of 10 °C min^−1^ under a nitrogen atmosphere from 100 to 1000 °C. The Raman spectra were collected using an WITEC‐ALPHA300R using a laser with an excitation wavelength of 532 nm (0.5 mW), focused on the sample with a 100x objective. The presented spectra were the mean over all the data acquired. The emission spectra of the light sources used for the photochemical reactions were recorded on an optical spectrometer StellarNet model Blue‐Wave UV‐NB50 (see Figure S1, Supporting Information). The reactor consisted of a custom‐made temperature‐controlled system, where the reaction mixture was kept at room temperature by passing a coolant through the metallic system employing a recirculating chiller, and the irradiation was achieved with a single LED located 1 cm beneath the base of the vial.

##### [4 + 2] Photofunctionalization of Carbon Nanotubes

In a typical experiment,^[^ ^42^ ^]^ if not otherwise stated, a vial was charged with a magnetic stirring bar, 3 mg of nanotubes **SWNT‐09**, **SWNT‐14**, or **MWNT**, 0.1 mmol of the aryl cyclobutyl amine substrate **3** and 0.01 mmol of photocatalyst in 1.5 mL of *o*‐dichlorobenzene (ODB) as solvent were used. The vial was sealed and degassed by 3 N_2_ freeze–pump–thaw cycles. The reaction was irradiated under white LED illumination at 20 °C for appropriate time, typically 40 h (Figure S1 and S2, Supporting Information). Then, the reaction vessel was opened, and the suspension was filtered through a 0.45 μm polytetrafluoroethylene (PTFE) membrane. The black powder was collected, suspended in 20 mL of dichloromethane (DCM), sonicated for 1 min, and filtered again. This washing procedure was repeated with DCM (4 × 20 mL), methanol (3 × 20 mL), and finally with acetone (3 × 20 mL). Drying under vacuum produced the corresponding sample **SWNT‐09‐3x**, **SWNT‐14‐3x**, and **MWNT‐3x**, where x stands for the different substrates employed.

## Supporting Information

Supporting Information is available from the Wiley Online Library or from the author.

## Conflict of Interest

The authors declare no conflict of interest.

## Supporting information

Supplementary Material

## Data Availability

The data that support the findings of this study are available in the supplementary material of this article.
