# Supplementary material for: Versatile Photofunctionalization of Carbon Nanotubes via [4 + 2] Cycloaddition: A Facile Route to Hybrid Nanomaterials
Source: Small Sci. 2025 Aug 31;5(11):2500191. doi: 10.1002/smsc.202500191 (PMC12622416; doi:10.1002/smsc.202500191)
Supplement: Supplementary file 1 — Supplementary Material [file SMSC-5-2500191-s001.pdf]

Supporting Information

Photochemical Covalent [4+2] Functionalization of Carbon Nanotubes

Giacomo de Crescenzo, Paula Sánchez-Morena, Alberto Fraile, Matías Blanco* and José Alemán*

Summary

S1. Extended synthesis

S2. Supporting Characterization data

S3. Organic molecules characterization spectra

S4. References

S1. EXTENDED SYNTHESIS

Synthesis of aryl cyclobutyl amines 3

Compounds **3** were Synthesized in a Buchwald-Hartwig amination protocol adapted from previously described methods, as described in table S1.[1]

Table S1. Synthesis of compounds **3**.[a]

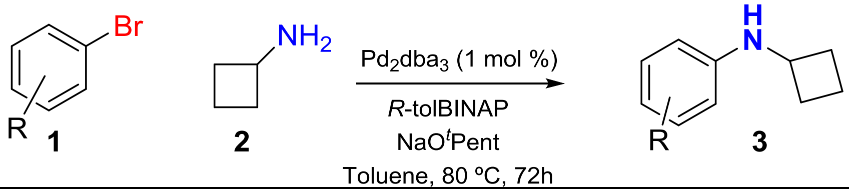

Entry	Precursor	Product	Yield[b]
1	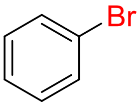 **1a**	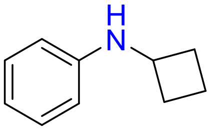 **3a**	64
2	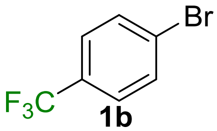 **1b**	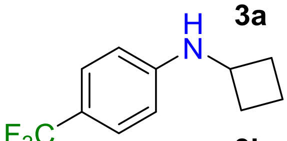 **3b**	70
3	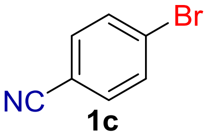 **1c**	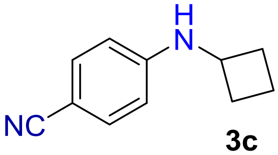 **3c**	59
4	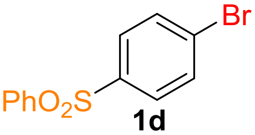 **1d**	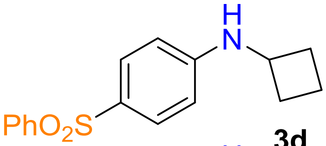 **3d**	41
5	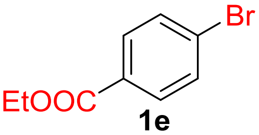 **1e**	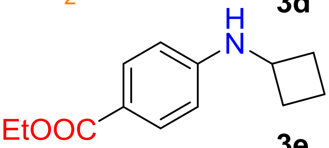 **3e**	62
6	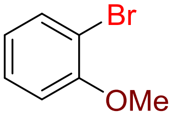 **1f**	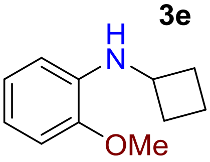 **3f**	58

[a] Reaction conditions: 2 mmol of **1** and 2.2 mmol of **2**, with Pd_2dba_3 and *R*-tolBINAP (1 mol%), using 3 mmol of base NaO^{t}Pent, in toluene (0.25 M) under inert atmosphere, at 80 ^{o}C for 72h. [b] Isolated yield (%)

In general, compounds **3** were synthesized as follows: In a sealed tube which contained 0.02 g (0.02 mmol) of tris(dibenzylideneacetone)dipalladium(0) and 0.01 g (0.02 mmol) of (*R*)-Tol-Binap, 2 mmol of aryl bromide **1** and 0.28 g (2.2 mmol) of cyclobutyl amine **2** were dissolved under inert atmosphere in 8 mL of degassed toluene. The mixture was stirred at room temperature for 15 min, and then 0.33 g (3 mmol) of sodium *tert*-pentoxide was added. The reaction was heated at 80 ^{o}C for 3 days. Then, it was cooled down, diluted with more toluene and filtered over a pad of celite. The pad was washed with several portions of toluene (4x10 mL), and the obtained filtrated was concentrated under vacuum.

Purification by flash chromatography (Silica, Cyclohexane (CyH) : Ethyl Acetate (AcOEt)) afforded the final product.

***N*-cyclobutylaniline (3a).** Synthesized following the general protocol starting from phenyl bromide (**1a**) that, after purification using as eluent CyH:AcOEt 96:4, yielded a yellow oil in 64 % yield. ^{1}H NMR (300 MHz, $CDCl_3$): δ 7.17 (t, *J* = 7.6 Hz, 2H), 6.70 (t, *J* = 7.2 Hz, 1H), 6.57 (d, *J* = 7.0 Hz, 2H), 3.91 (dd, J = 14.6, 7.3 Hz, 1H), 3.87 (bs, 1H) 2.43 (m, 2H), 1.82 (m, 4H). This compound was described previously in literature and matched with the obtained data.[1]

***N*-cyclobutyl-4-(trifluoromethyl)aniline (3b)**. Synthesized following the general protocol starting from 4-(α,α,α,-trifluoro)phenyl bromide (**1b**) that, after purification using as eluent CyH:AcOEt 95:5, yielded a yellow oil in 70 % yield. ^{1}H NMR (300 MHz, $CDCl_3$): δ 7.44 (d, *J* = 8.3 Hz, 2H), 6.59 (d, *J* = 8.3 Hz, 2H), 4.21 (bs, 1H), 4.11 – 3.91 (m, 1H), 2.60 – 2.38 (m, 2H), 1.99 – 1.83 (m, 4H). ^{13}C NMR (125 MHz, $CDCl_3$): δ 149.7, 126.6 (q, *J* = 3.9 Hz), 125.1 (q, *J* = 270.0 Hz), 118.6 (q, *J* = 32.6 Hz), 112.0, 48.5, 31.0, 15.3. This compound was described previously in literature and matched with the obtained data.[1]

4-(cyclobutylamino)benzonitrile (3c). Synthesized following the general protocol starting from 4-cyanophenyl bromide (**1c**) that, after purification using as eluent CyH:AcOEt 95:5, yielded a white solid in 59 % yield. ^{1}H NMR (300 MHz, $CDCl_3$): δ 7.39 (d, *J* = 8.1 Hz, 2H), 6.49 (d, *J* = 8.2 Hz, 2H), 4.37 (bs, 1H), 3.90 (dt, *J* = 14.2 Hz, 7.1 Hz, 1H, 2.55 – 2.32 (m, 2H), 1.92 – 1.79 (m, 4H). ^{13}C NMR (75 MHz, $CDCl_3$): δ 150.33, 133.66, 120.61, 112.32, 98.37, 48.19, 30.82, 15.29. MS (ESI): (m/z): 173.2 $(M+H)^+$.

***N*-cyclobutyl-4-(phenylsulfonyl)aniline (3d).** Synthesized following the general protocol starting from 4-(phenylsulfonyl)phenyl bromide (**1d**) that, after purification using as eluent CyH:AcOEt 90:10, yielded a white solid in 41 % yield. ^{1}H NMR (300 MHz, $CDCl_3$): δ 7.89 (d, *J* = 8.0, 2H), 7.76 – 7.64 (m, 3H), 7.50 – 7.42 (m, 2H), 6.50 (d, *J* = 8.1 Hz, 2H), 4.37 (bs, 1H), 4.02 – 3.84 (m, 1H), 2.53 – 2.36 (m, 2H), 1.93 – 1.78 (m, 4H). ^{13}C NMR (75 MHz, $CDCl_3$): δ 151.0, 143.3, 132.4, 129.8, 129.1, 127.5, 127.0, 112.0, 48.2, 30.8, 15.3. MS (ESI): (m/z): 288.2 $(M+H)^+$.

Ethyl 4-(cyclobutylamino)benzoate (3e). Synthesized following the general protocol starting from ethyl 4-bromobenzoate (**1e**) that, after purification using as eluent CyH:AcOEt 98:2, yielded a white-yellow solid in 62 % yield. ^{1}H NMR (300 MHz, $CDCl_3$): δ (d, *J* = 8.6 Hz, 2H), 6.54 (d, *J* = 8.6 Hz, 2H), 4.36 (q, *J* = 7.1 Hz, 2H), 4.09 – 3.95 (m, 1H), 2.58 – 2.38 (m, 2H), 2.02 – 1.77 (m, 4H), 1.41 (t, *J* = 7.1 Hz, 3H). This compound was described previously in literature and matched with the obtained data.[1]

***N*-cyclobutyl-2-methoxyaniline (3f).** Synthesized following the general protocol starting from 4-methoxyphenyl bromide (**1f**) that, after purification using as eluent CyH:AcOEt 95:5, yielded a yellow oil in 41 % yield. ^{1}H NMR (500 MHz, $CDCl_3$): δ 6.93 – 6.87 (m, 1H), 6.81 (dd, *J* = 7.9, 1.4 Hz, 1H), 6.74 – 6.67 (m, 1H), 6.57 (dd, *J* = 7.8, 1.5 Hz, 1H), 4.39 (bs, 1H), 3.88 (s, 3H), 2.57 – 2.38 (m, 2H), 2.06 – 1.69 (m, 4H). ^{13}C NMR (125 MHz, $CDCl_3$): δ 146.8, 137.1, 121.3, 116.5, 110.4, 109.3, 55.4, 48.7, 31.2, 15.4. This compound was described previously in literature and matched with the obtained data.[1]

Purification of carbon nanotubes

Commercially available SWNT, both with 0.9 nm or 1.4 nm in diameter (50 mg) were treated with concentrated hydrochloric acid (30 mL), and the mixture was magnetically stirred at 60 °C for 2 h. After cooling down, the solid was isolated by centrifugation and washed with fresh Milli-Q water with the necessary centrifugation cycles until the supernatant reached neutral pH. After drying under vacuum, the procedure yielded the purified **SWNT-09** and **SWNT-14** sample.[2,3]

Synthesis of amino-enriched organic polymer nanoparticles

In a round bottom flask equipped with a magnetic stirring bar, tetra(4-anilyl)methane (0.25 mmol, 0.095 g) and terephtaldehyde (0.45 mmol, 0.06 g) were dissolved in 30 mL of dichloromethane (DCM). To this solution, 3 mL of acetic acid were added, and the mixture was magnetically stirred at room temperature for 24h. The yellow precipitate formed was then filtered and washed with fresh DCM. It was dried under vacuum to yield the sample **OP-1** (scheme S1).[4]

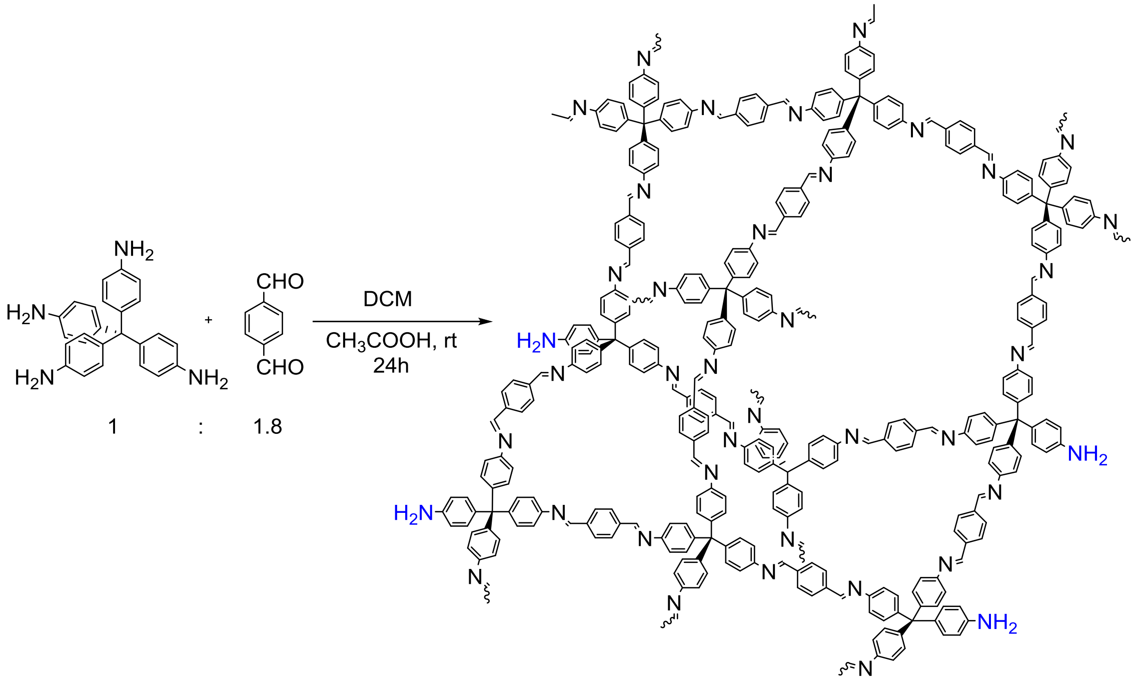

Scheme S1. Synthesis of **OP-1**.

Post-functionalization hydrolysis

9 mg of sample **SWNT-09-3e** was added to a reaction flask containing 9 mL of 1 M aqueous solution of KOH. The mixture was magnetically stirred at 100 ^{o}C for 16 h (Scheme S2). After such time, the system was allowed to cool to room temperature and was centrifuged. The supernatant was discarded, and the solid powder was washed with enough number of centrifugation cycles till the supernatant reached neutral pH. Drying under vacuum afforded sample **SWNT-09-3e-B**.[5]

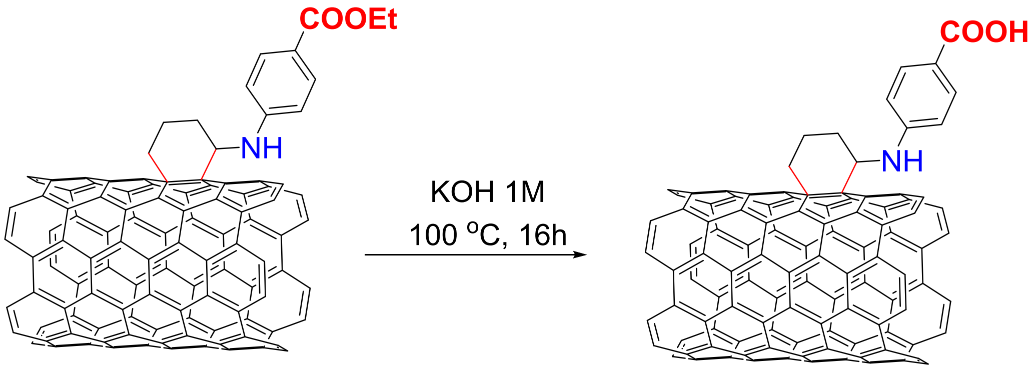

Scheme S2. Post-functionalization hydrolysis.

Synthesis of the hybrid material through carbodiimide coupling

5 mg of sample **SWNT-09-3e-B** was suspended in DMF though sonication to form a stable suspension with a concentration of 1 mg mL^{-1}. To this suspension, 8 mg (0.05 mmol) of hydroxybenzotriazole (HOBt) and 6 µL (0.05 mmol) of triethylamine (TEA) were added. The mixture was kept under stirring for 45 min at 0 °C in an ice bath. Then, the ice bath was removed, and 10 mg (0.05 mmol) of *N*-(3-Dimethylaminopropyl)-*N'*-ethylcarbodiimide hydrochloride (EDC) and 1 mg amino-enriched organic polymer were added. The reaction mixture was kept under stirring at room temperature for 6 days. The resulting solid was separated by centrifugation and thoroughly washed with 3 × 15 mL of fresh DMF, 3 × 15 mL of a 1:1 DMF:water mixture, 3 × 15 mL of methanol, and 2 × 15 mL of acetone. After each centrifugation step, the powder was suspended in the fresh washing solvent and sonicated for 5 min in order to remove the eventual physisorbed species. Finally, the sample was dried under vacuum.[6]

Functionalization degree estimation

In samples **CNT-3a** (Scheme S3), the motif has a weight very similar to molecule **3a**: MW = 146. Using stoichiometric calcutations, we can estimate the $mmol_{\mathbf{3a}}$/g of material using the weight loss detected by TGA assuming that all the organic molecule covalently bonded to the nanotube is thermally degraded below 400 ^{o}C. We can also run this stoichoimetric calculation for the elemental analysis assuming that all N detected in the sample corresponds to the aryl-cyclobutyl amine covalently bonded.

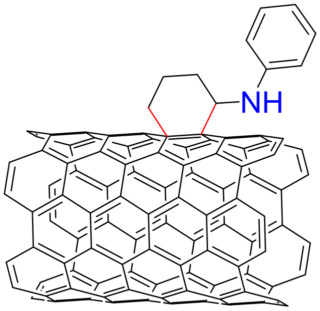

CNT-3a

	TGA/EA	FG
**SWNT-09-3a**	14	1.1
	2.4	1.4
**SWNT-14-3a**	12	0.8
	1.4	0.7
**MWNT-3a**	8.0	0.5
	1.6	0.8

Scheme S3. Functionalization degree estimation

S.2 SUPPORTING CHARACTERIZATION DATA

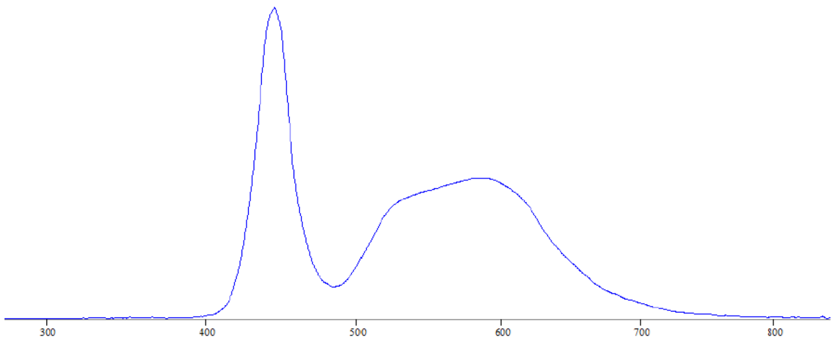

Figure S1. Emission spectrum of 40 mW white LED of the custom-made temperature-controlled system.

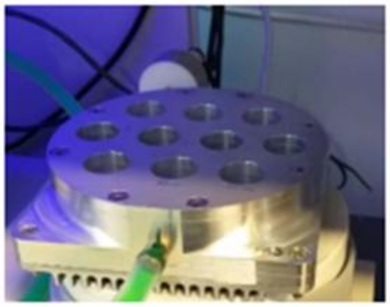

Figure S2. Digital image of the reactor

Table S2. Elemental analysis of **SWNT-09-3a** samples resulting from the [4+2] photocycloaddition optimization.

Variation	C	H	N	S
Ir catalyst	77.3	3.8	0.9	0.5
Ir catalyst (**SWNT-14**)	81.2	3.5	0.7	0.7
Ir catalyst (**MWNT**)	83.8	2.0	0.9	0.1
No catalyst	79.2	3.2	0.6	0.5
No light	79,5	3,2	0,5	0,1
Eosin Y	76.0	2.7	1.9	1.0
Rose Bengal	76.1	2.7	1.3	1.4
Rhodamine 6G	71.3	3.3	2.1	0.8
Rhodamine B	72.0	3.8	2.3	0.3
Values reported in % wt.				

Table S3. Elemental analysis of samples resulting from the optimized [4+2] photocycloaddition and further reactions.

Sample	C	H	N	S	S/N[a]
**SWNT-09**	77.5	1.8	0.3	0.1	0.3
**SWNT-09-3a**	72.0	3.8	2.3	0.3	0.1
**SWNT-09-3b**	78.8	2.8	1.4	0.2	0.1
**SWNT-09-3c**	78.6	3.1	2.2	0.1	0.0
**SWNT-09-3d**	75.5	3.4	1.3	1.5	1.1

**SWNT-09-3e**	79.5	3.0	1.8	0.0	0
**SWNT-09-3e-B**	79.1	2.8	1.6	0.1	0.0
**SWNT-09-3f**	75.4	3.0	1.3	0.1	0.0
**SWNT-14**	83.1	1.4	0.4	0.1	0.2
**SWNT-14-3a**	81.7	2.9	1.4	0.4	0.3
**MWNT**	86.2	0.5	0.1	0.0	0
**MWNT-3a**	81.5	1.6	1.3	0.3	0.2
**OP-1**	79.1	5.3	8.7	0.1	0.0
**SWNT-OP1**	80.7	3.7	2.3	0.1	0.0
Values reported in % wt. a) ratio between the amount of S and N					

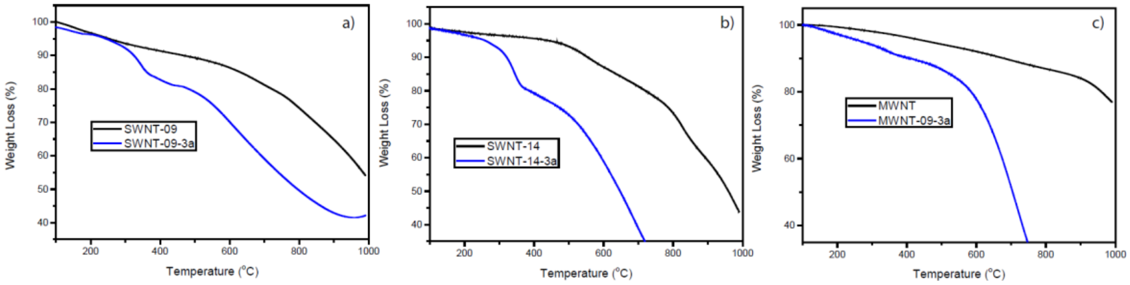

Figure S3. TGA curves of samples a) **SWNT-09-3a**, b) **SWNT-14-3a** and c) **MWNT-3a** compared to their corresponding pristine nanotubes.

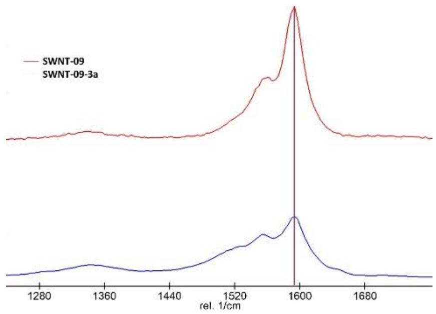

Figure S4. Raman D-G band region of sample **SWNT-09** and **SWNT-09-3a** resulting from the [4+2] cycloaddition.

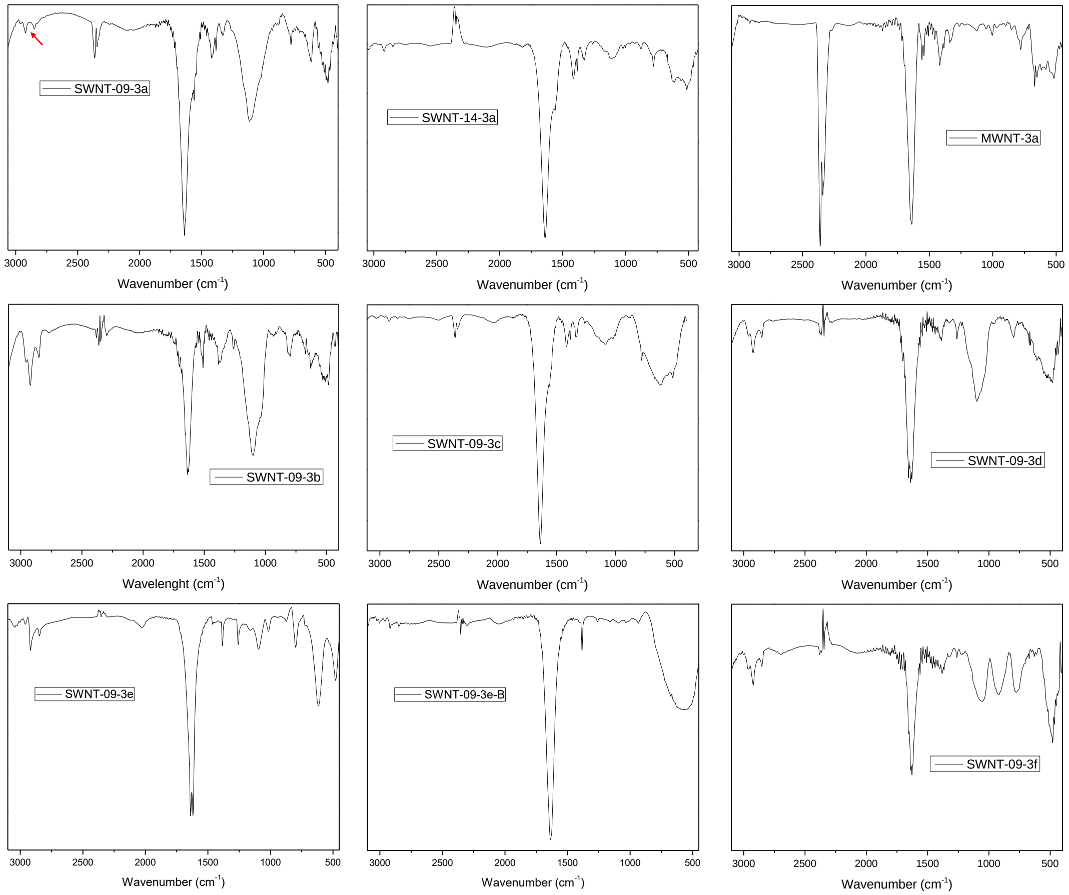

Figure S5. FTIR spectra of samples resulting from the [4+2] cycloaddition and further reactions.

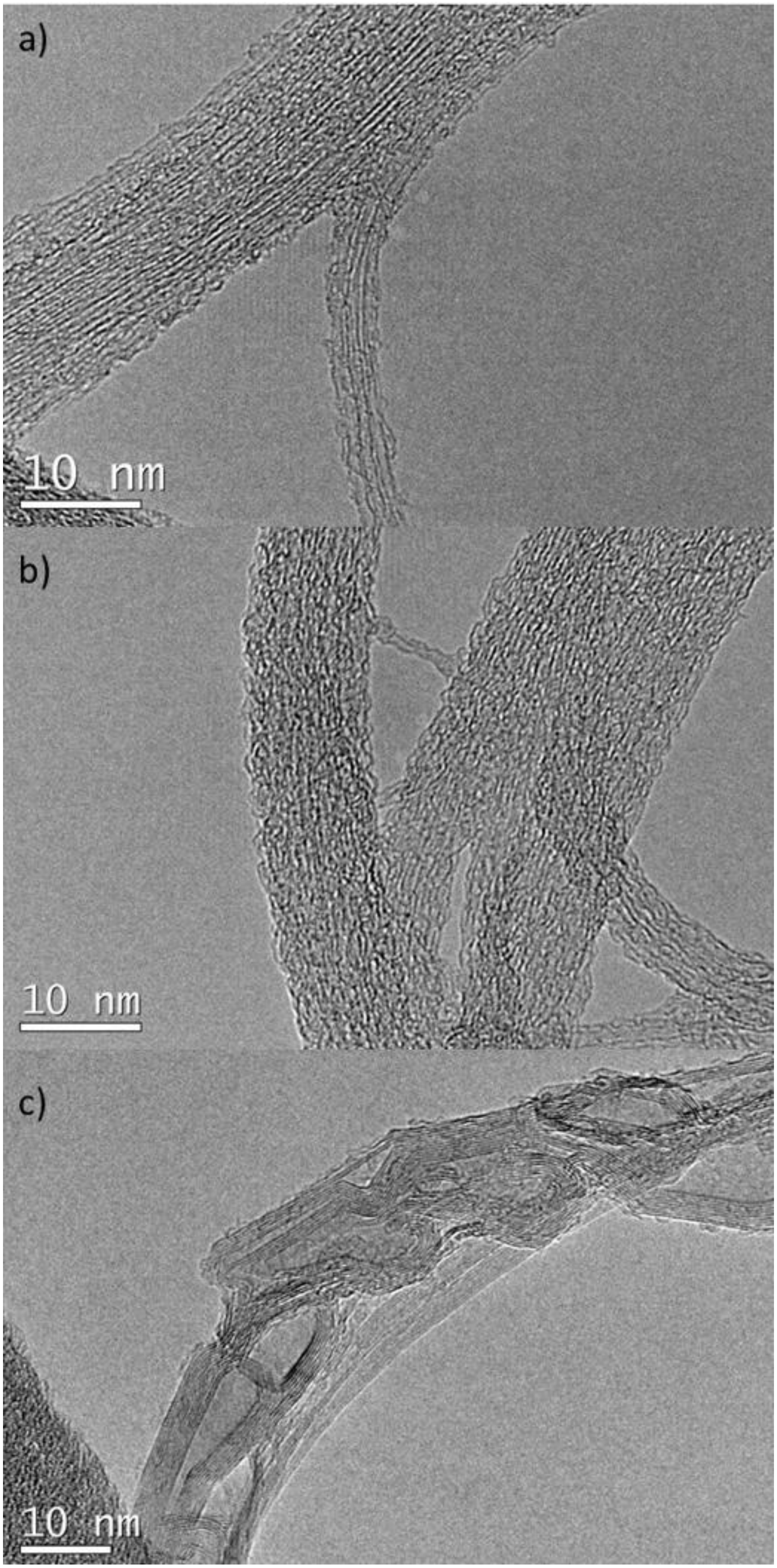
a)
10 nm
b)
10 nm
c)
10 nm

Figure S6. TEM images of pristine materials a) **SWNT-09**, b) **SWNT-14** and c) **MWNT.**

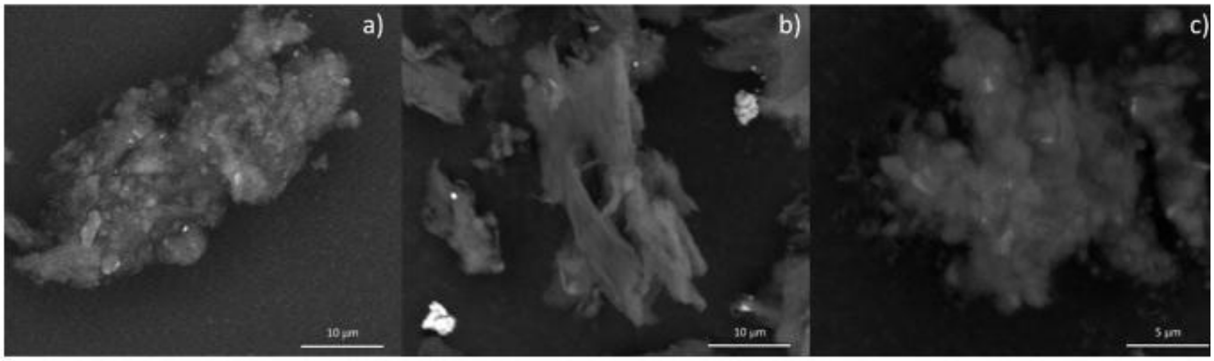

Figure S7. SEM images of pristine materials a) **SWNT-09**, b) **SWNT-14** and c) **MWNT.**

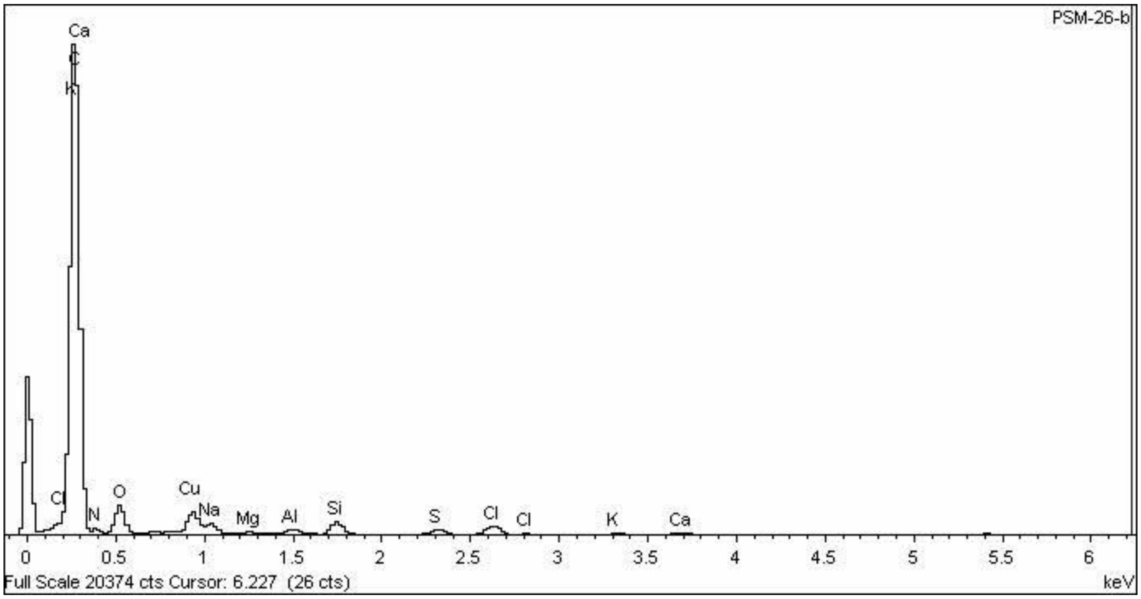

Figure S8. EDX Spectrum of sample **SWNT-09-3a.**

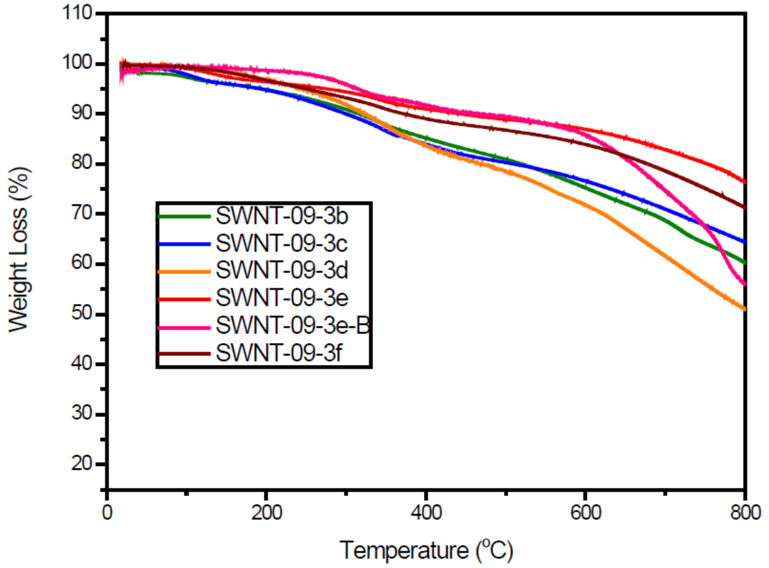

Figure S9. TGA curves of samples **SWNT-09-3b**, **SWNT-09-3c**, **SWNT-09-3d, SWNT-09-3e, SWNT-09-3e-B** and **SWNT-09-3f.**

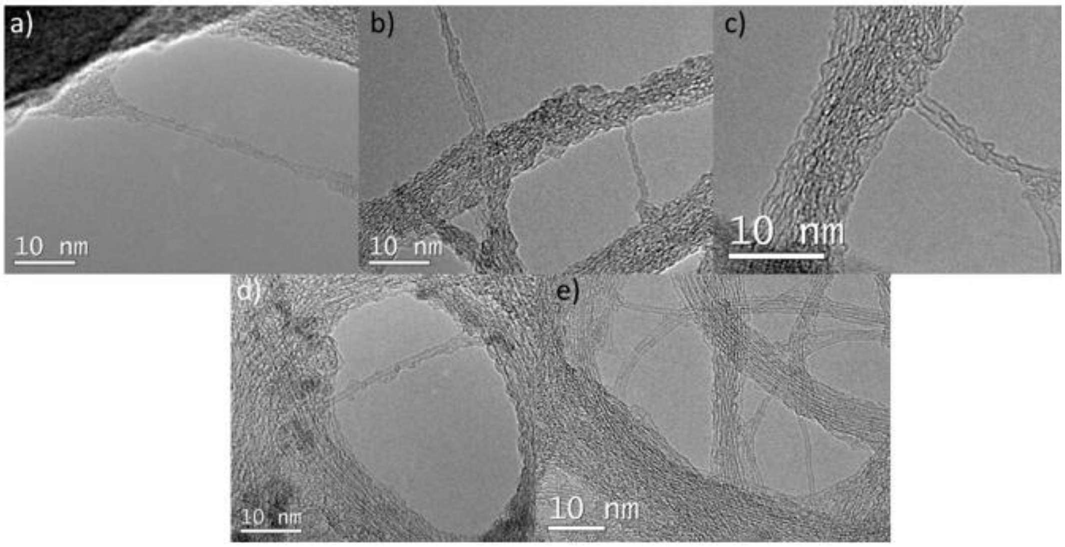

Figure S10. Additional TEM images of samples a) **SWNT-09-3b**, b) **SWNT-09-3c**, c) **SWNT-09-3d**, d) **SWNT-09-3e** and e) **SWNT-09-3f**

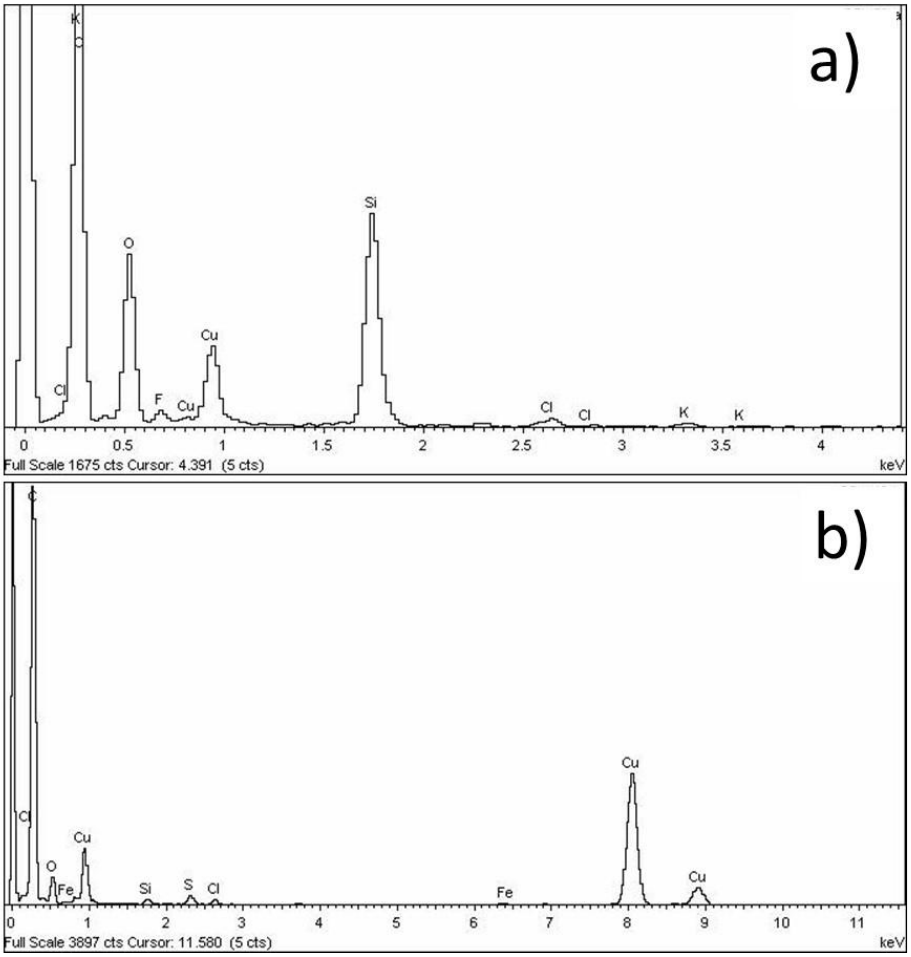

Figure S11. EDX spectra of samples a) **SWNT-09-3b** and **SWNT-09-3d.**

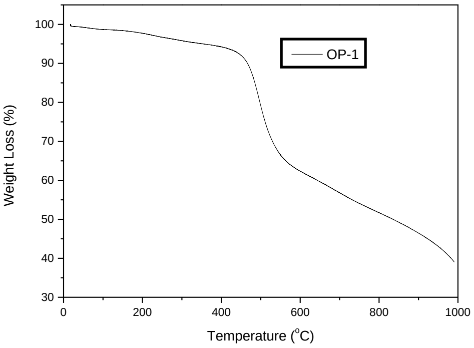

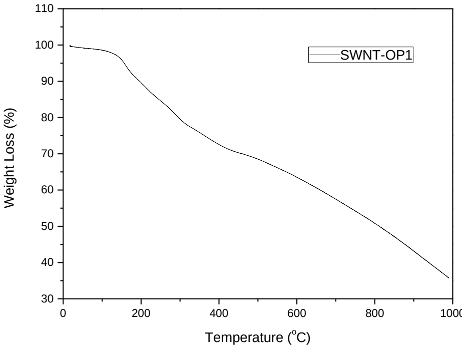

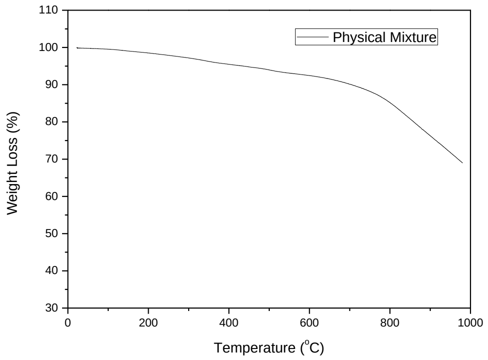

Figure S12. TGA curves of **OP-1**, **SWNT-OP1** and their physical mixture.

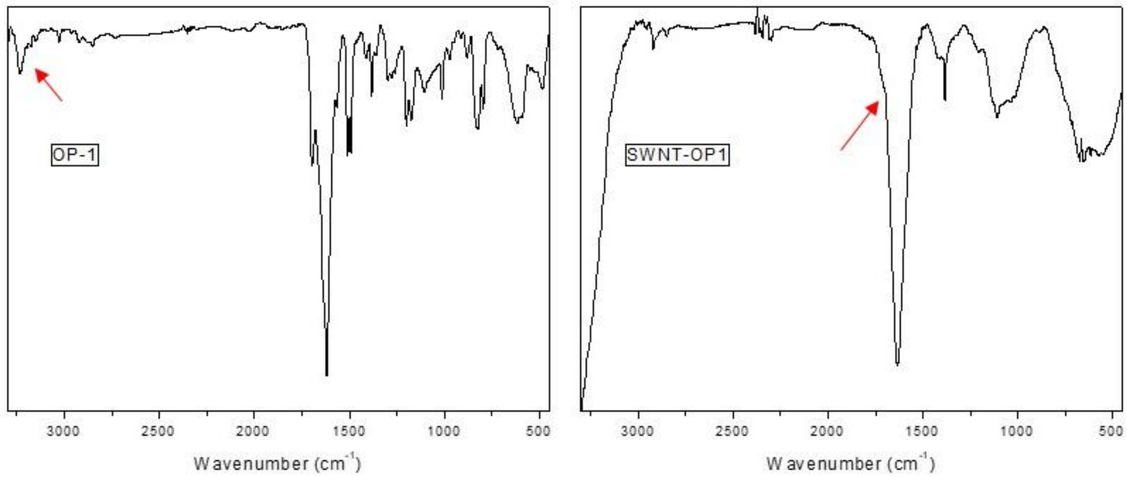

Figure S13. FTIR OP-1 and **SWNT-OP1**

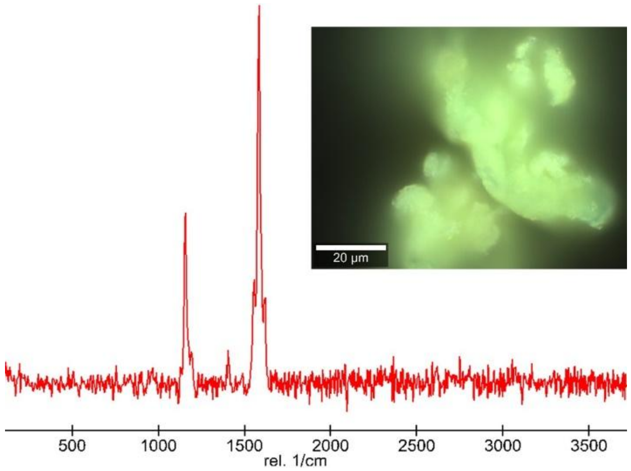

Figure S14. Raman spectrum and optical image of **OP-1.**

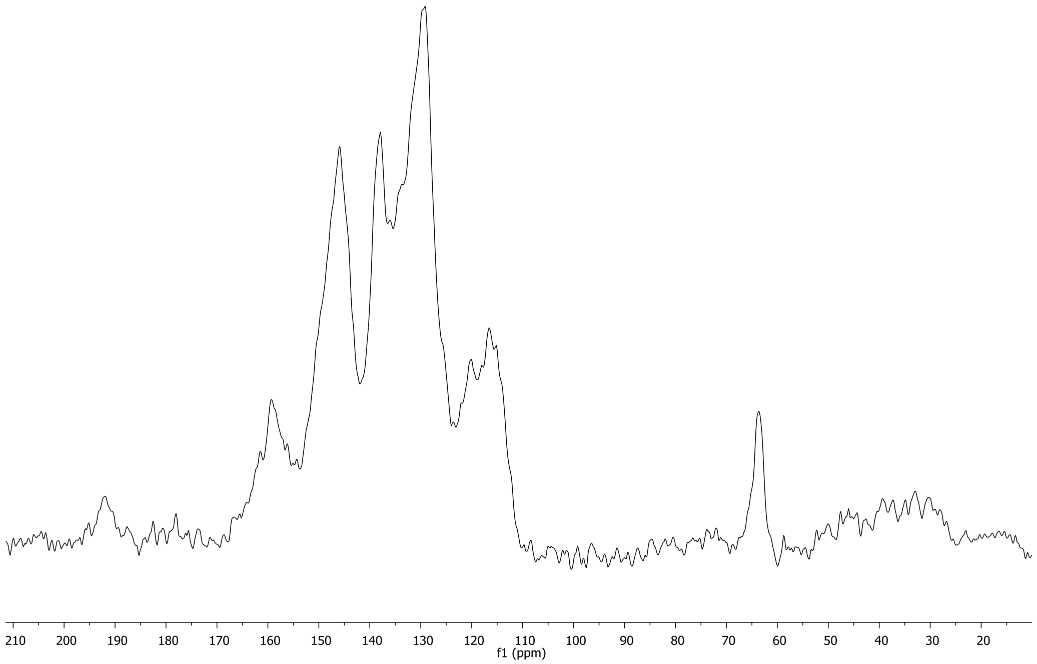

Figure S15. ^{13}C-SSNMR spectrum of sample **OP-1**.

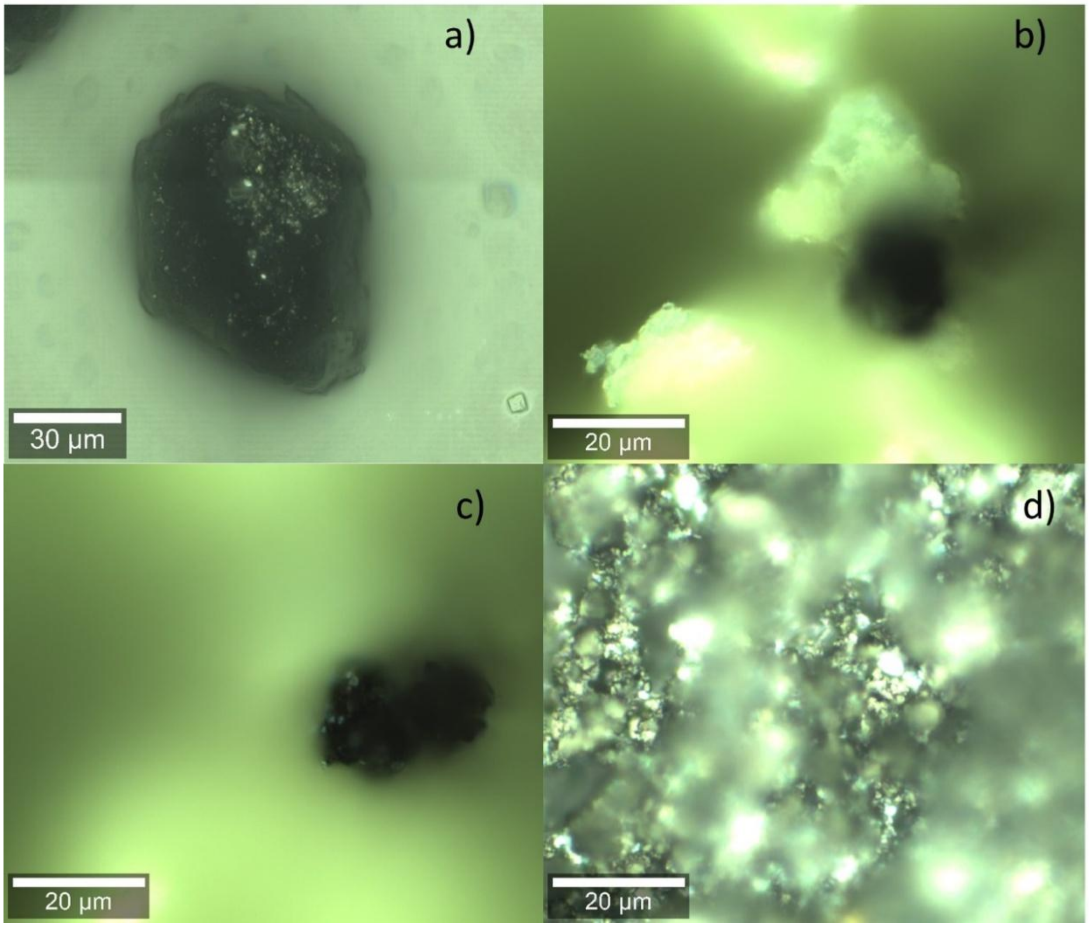

Figure S16. Optical images of a) **SWNT,** b) and c) the composite and d) **SWNT-OP1.**

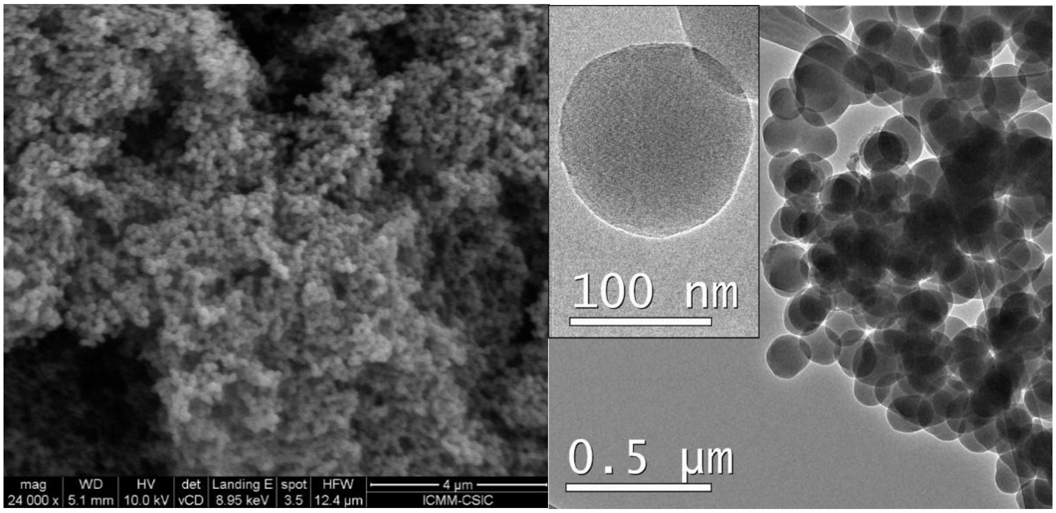

Figure S17. SEM and TEM images of **OP-1**

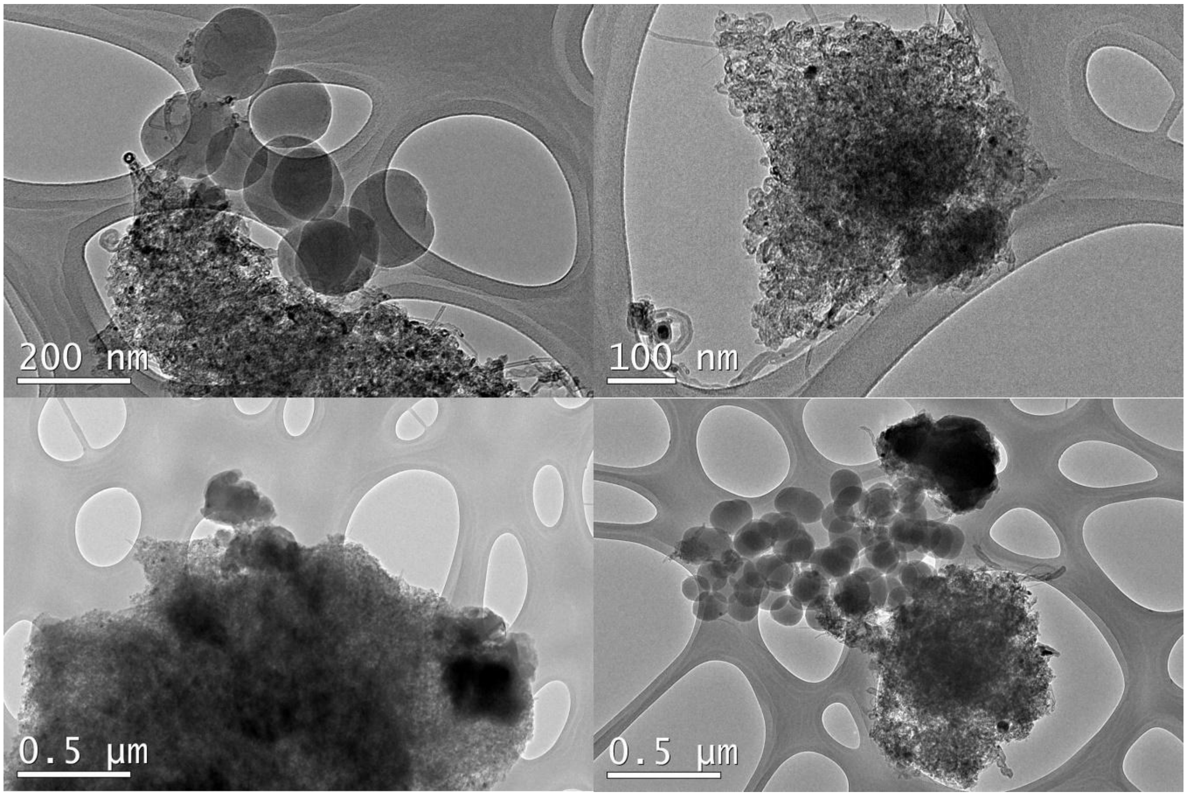

Figure S18. Additional TEM images of **SWNT-OP1**

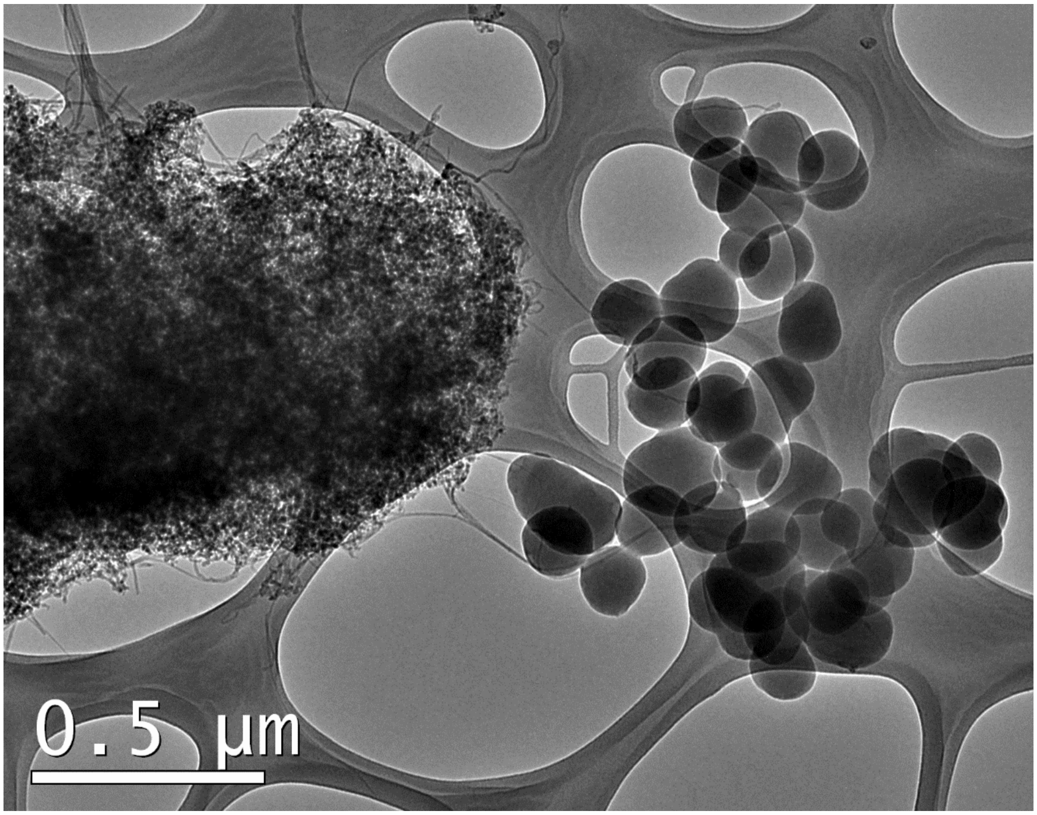

Figure S19. TEM image of the composite between **SWNT** and **OP-1.**

S3. ORGANIC MOLECULES CHARACTERIZATION SPECTRA

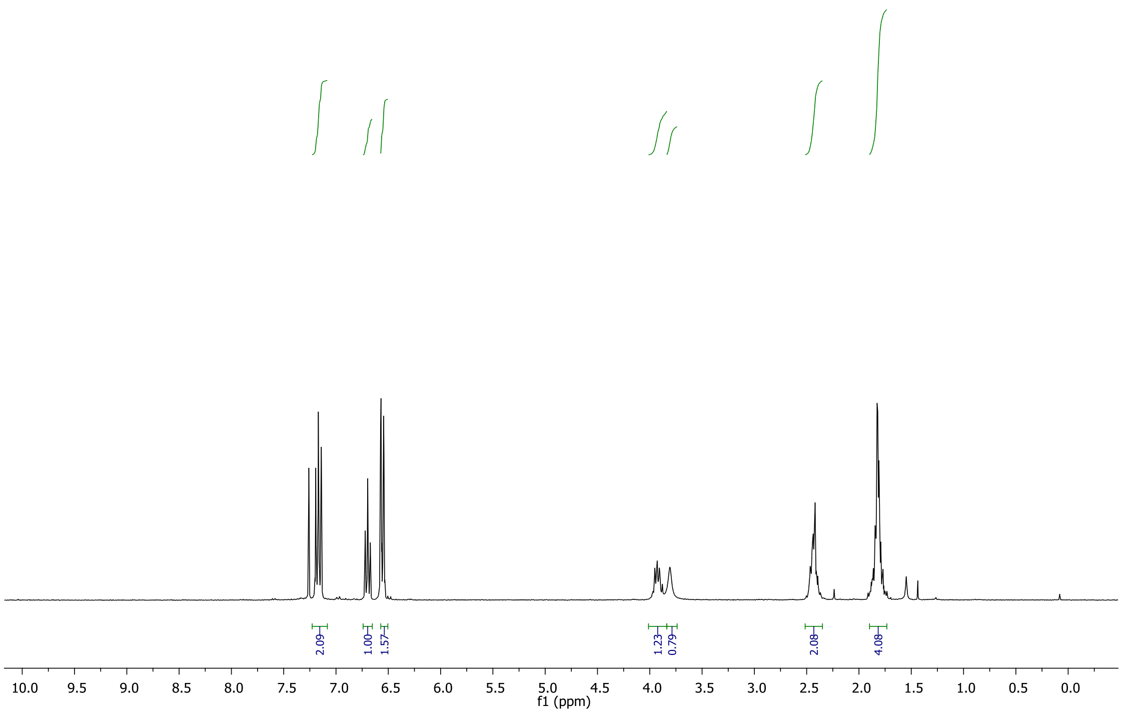

Figure S20. ^{1}H-NMR spectrum ($CDCl_3$, 300 MHz, 298 K) of compound **3a.**

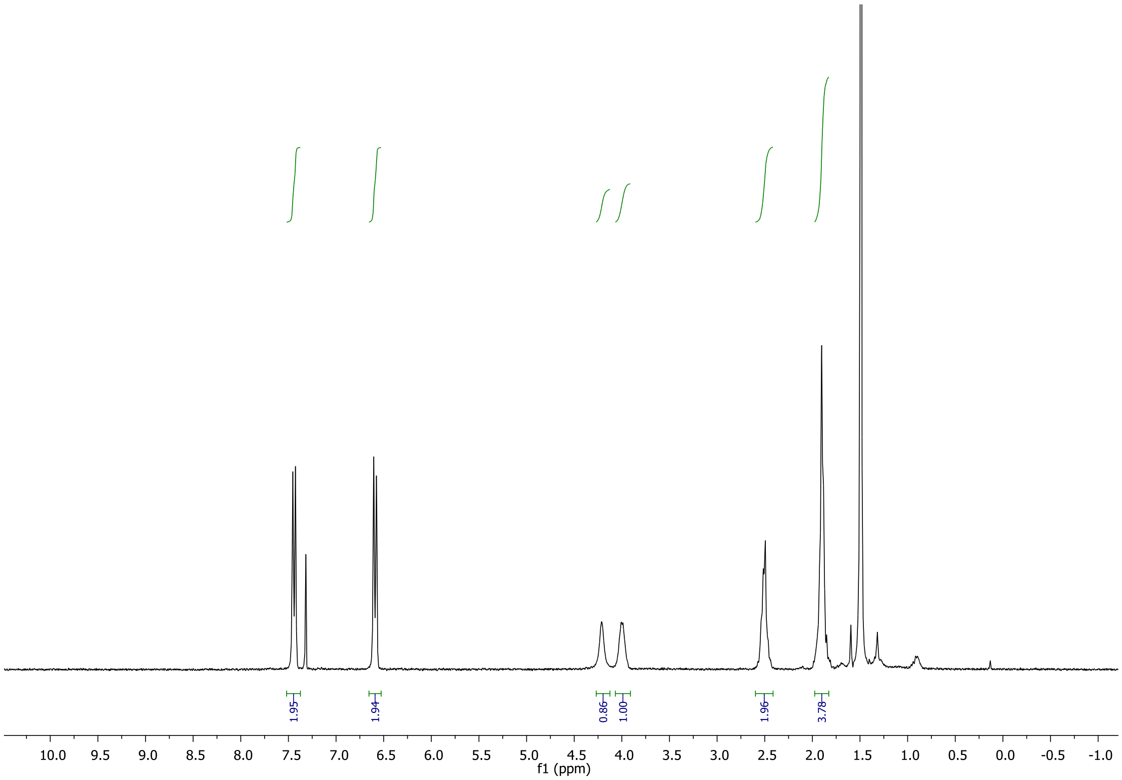

Figure S21. ^{1}H-NMR spectrum ($CDCl_3$, 300 MHz, 298 K) of compound **3b.**

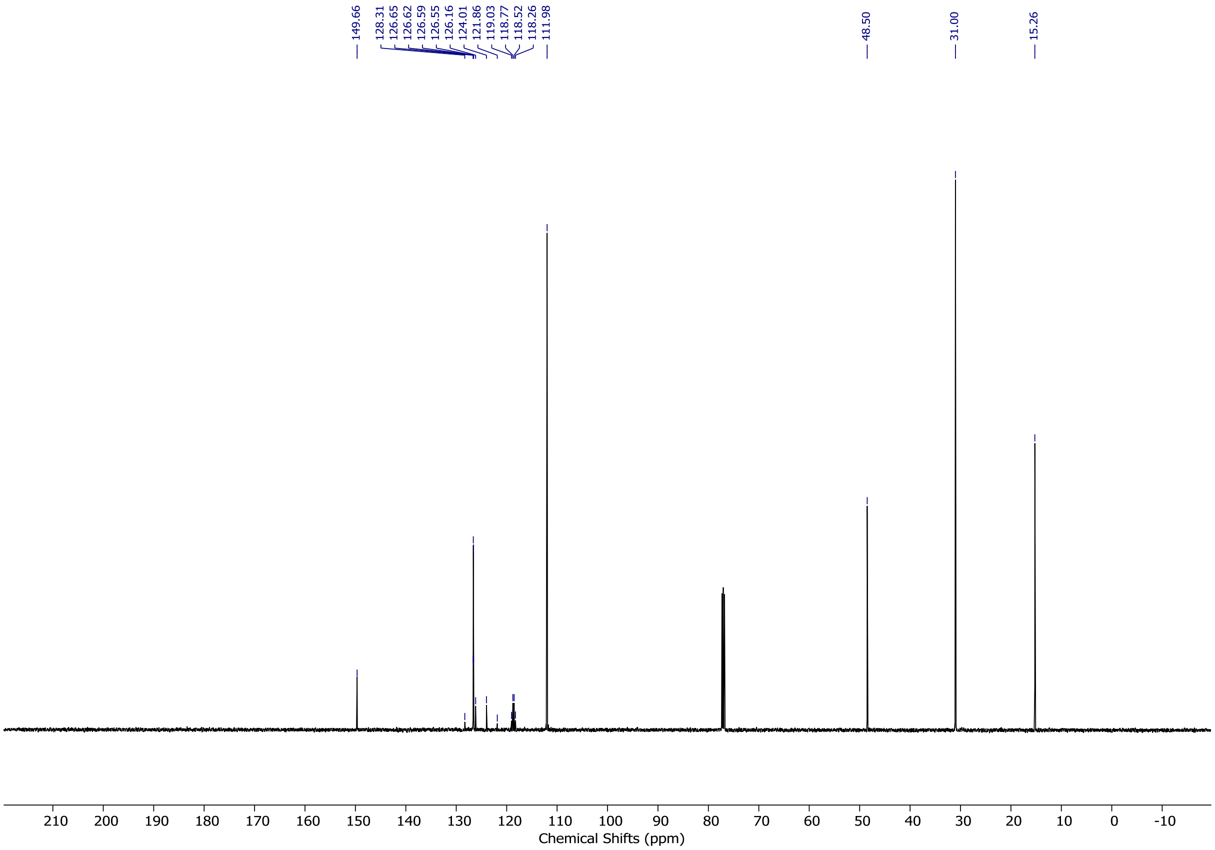

Figure S22. ^{13}C-NMR spectrum ($CDCl_3$, 125 MHz, 298 K) of compound **3b.**

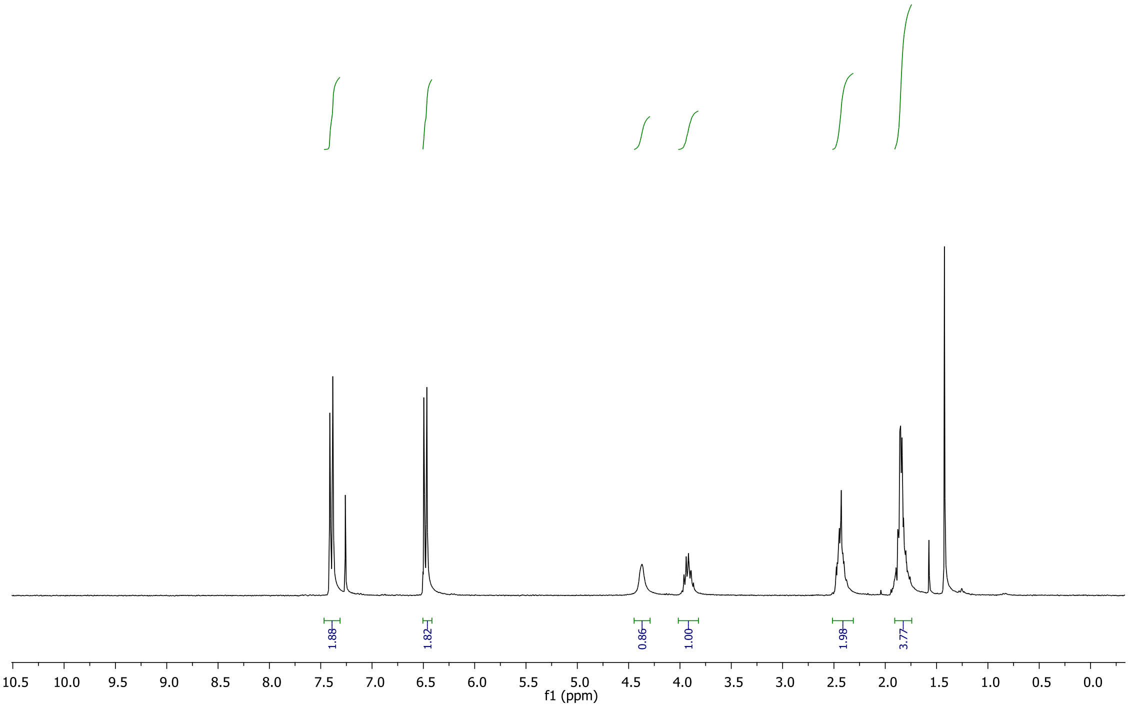

Figure S23. ^{1}H-NMR spectrum ($CDCl_3$, 300 MHz, 298 K) of compound **3c.**

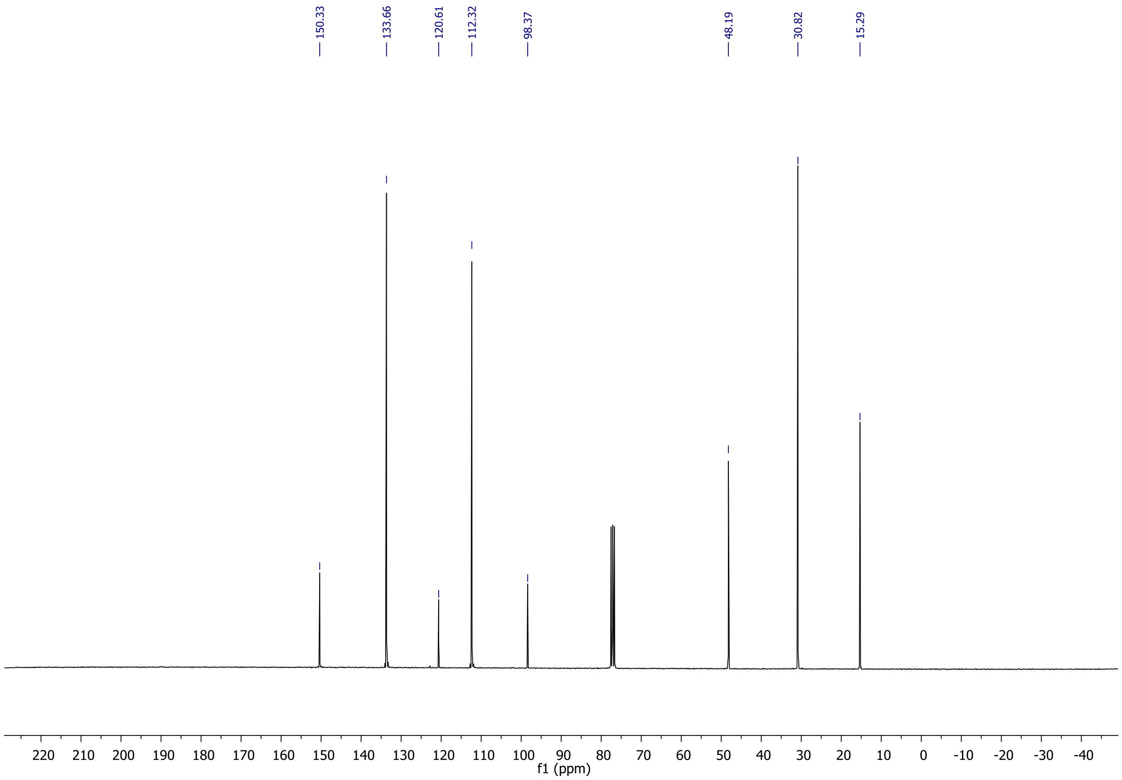

Figure S24. ^{13}C-NMR spectrum ($CDCl_3$, 75 MHz, 298 K) of compound **3c.**

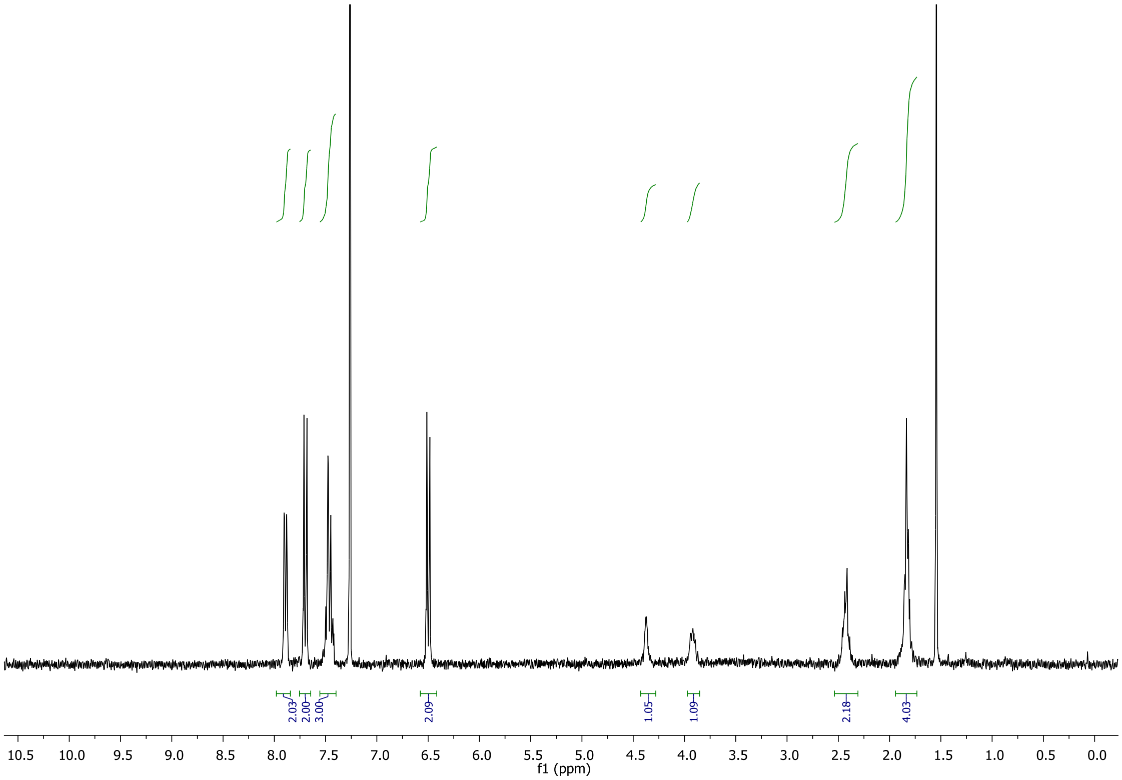

Figure S25. ^{1}H-NMR spectrum ($CDCl_3$, 300 MHz, 298 K) of compound **3d.**

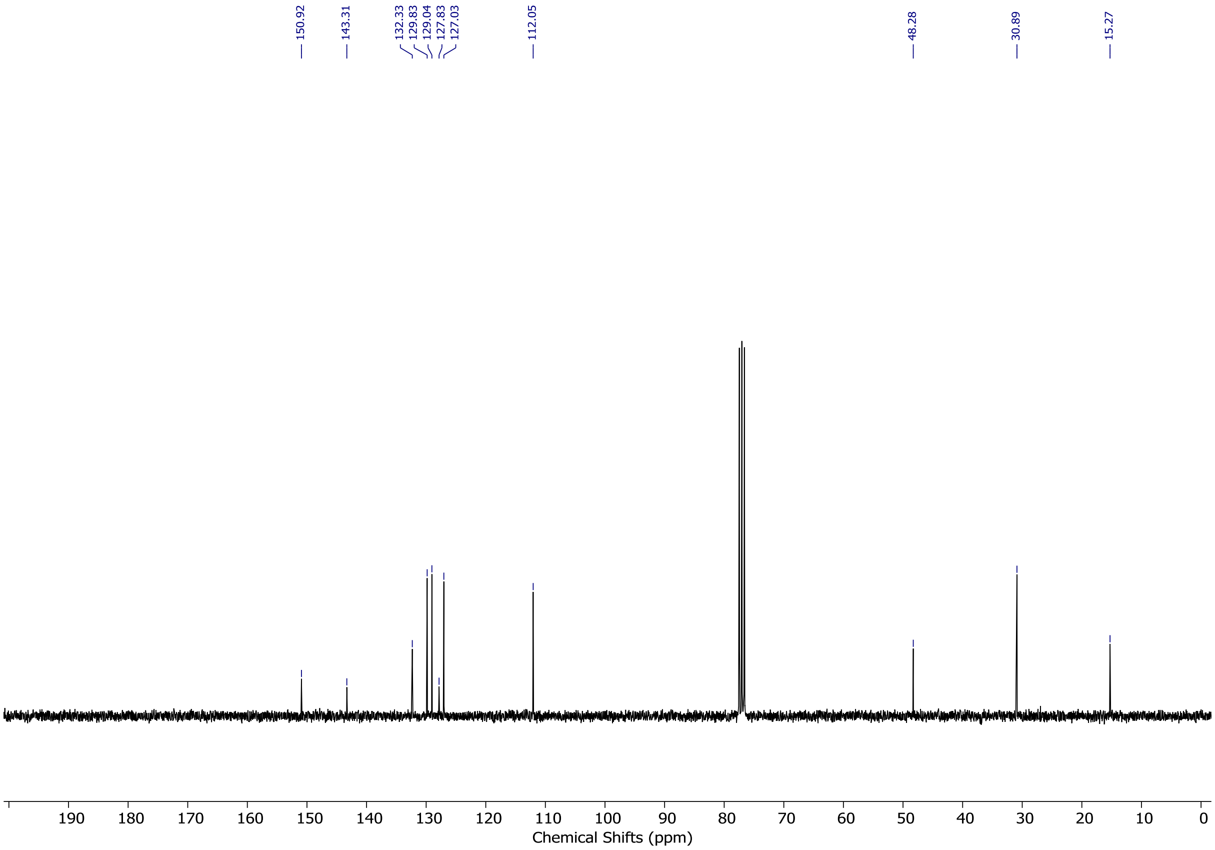

Figure S26. ^{13}C-NMR spectrum ($CDCl_3$, 75 MHz, 298 K) of compound **3d.**

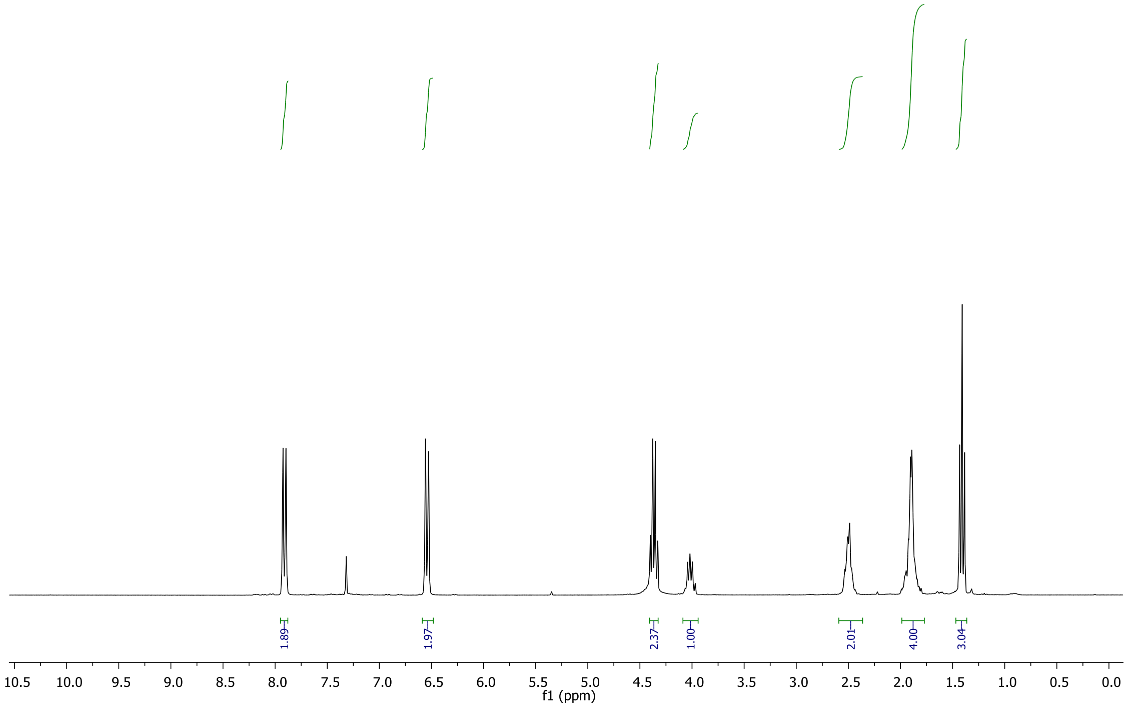

Figure S27. ^{1}H-NMR spectrum ($CDCl_3$, 300 MHz, 298 K) of compound **3e.**

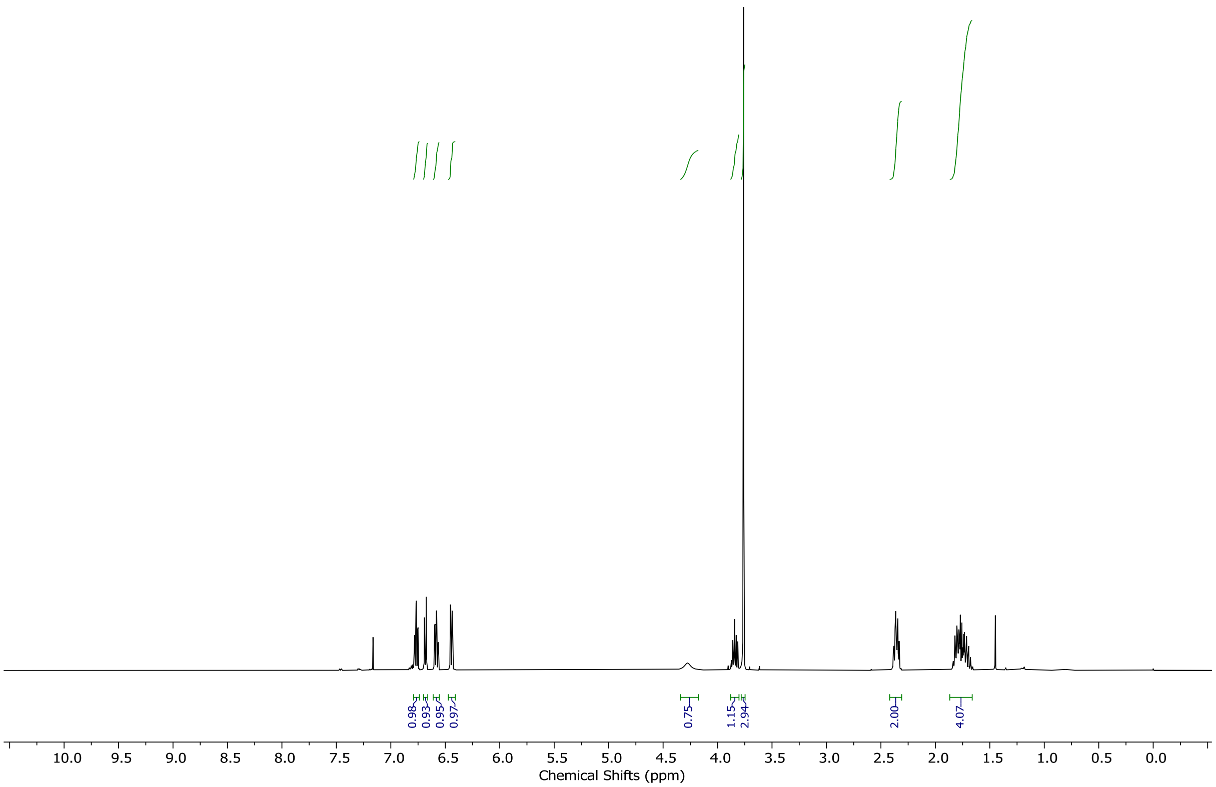

Figure S28. ^{1}H-NMR spectrum ($CDCl_3$, 500 MHz, 298 K) of compound **3f.**

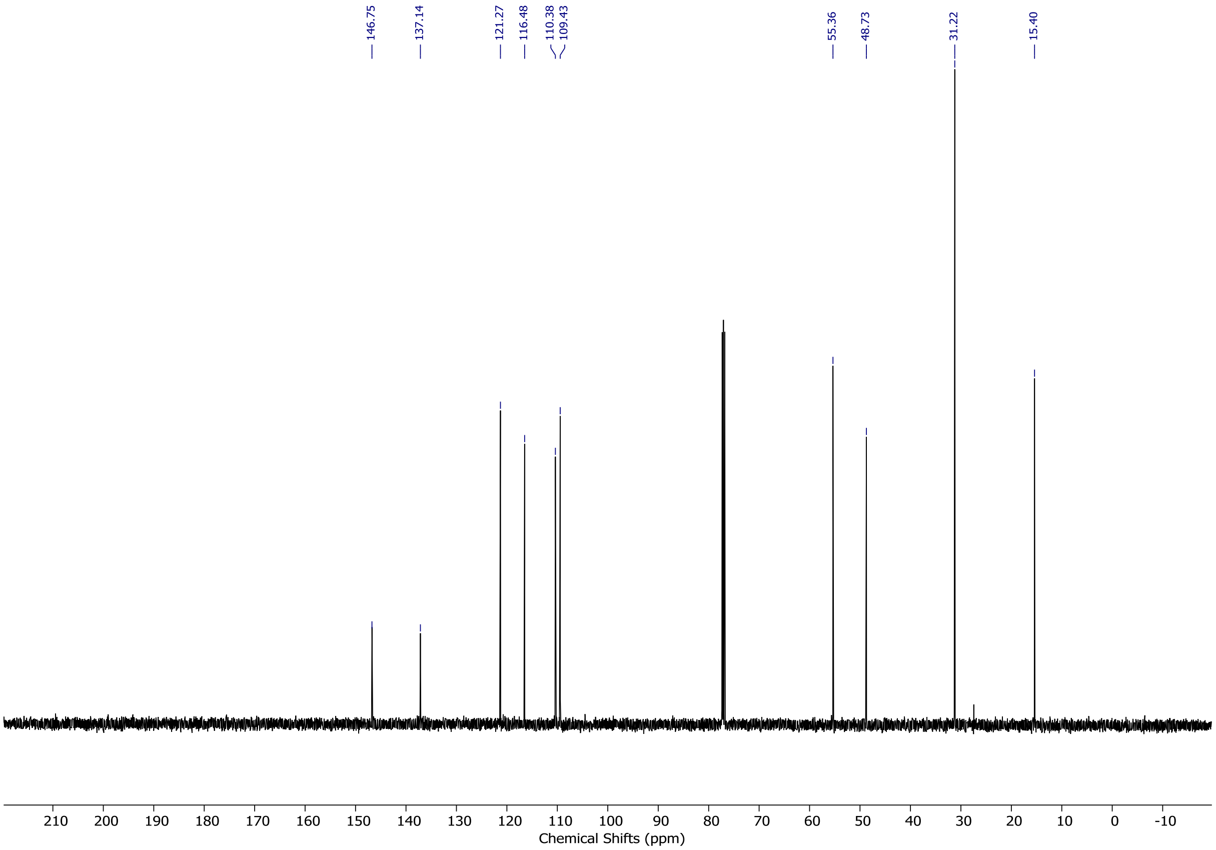

Figure S29. ^{13}C-NMR spectrum ($CDCl_3$, 125 MHz, 298 K) of compound **3f.**

S4. REFERENCES

[1] J. Wang, N. Zheng. *Angew. Chem. Int. Ed.* **2015**, *54*, 39, 11424–11427.

[2] M. Blanco, P. Álvarez, C. Blanco, N. Campos, D. Gómez, R. Menéndez. *Diamond and related materials* **2013,** *37*, 1-7.

[3] D. Gonzalez-Muñoz, A. Martín-Somer, K. Strobl, S. Cabrera, P. J. De Pablo, S. Díaz-Tendero, M. Blanco, J. Alemán. *ACS Appl. Mater. Interfaces* **2021**, *13*, 24877-24886.

[4] A. Jiménez-Almarza, A. López-Magano, L. Marzo, S. Cabrera, R. Mas-Ballesté, J. Alemán. *Chemcatchem* **2019,** *11*, 4916-4922.

[5] M. Ferrándiz-Saperas, A. Ghisolfi, D. Cazorla-Amorós, C. Nájera, J. M. Sansano, *Chem. Commun*. **2019**, *55*, 7462-7465.

[6] B. Ma, M. Blanco, L. Calvillo, L. Chen, G. Chen, T.-C. Lau, G. Dražić, J. Bonin, M. Robert, G. Granozzi, *J. Am. Chem. Soc.* **2021**, *143*, 8414-8425.
